# Gene Expression Networks Underlying Ovarian Development in Wild Largemouth Bass (*Micropterus salmoides*)

**DOI:** 10.1371/journal.pone.0059093

**Published:** 2013-03-20

**Authors:** Christopher J. Martyniuk, Melinda S. Prucha, Nicholas J. Doperalski, Philipp Antczak, Kevin J. Kroll, Francesco Falciani, David S. Barber, Nancy D. Denslow

**Affiliations:** 1 Department of Physiological Sciences and Center for Environmental and Human Toxicology, University of Florida, Gainesville, Florida, United States of America; 2 Canadian Rivers Institute and Department of Biology, University of New Brunswick, Saint John, New Brunswick, Canada; 3 Institute of Integrative Biology, University of Liverpool, Liverpool, United Kingdom; Karlsruhe Institute of Technology, Germany

## Abstract

**Background:**

Oocyte maturation in fish involves numerous cell signaling cascades that are activated or inhibited during specific stages of oocyte development. The objectives of this study were to characterize molecular pathways and temporal gene expression patterns throughout a complete breeding cycle in wild female largemouth bass to improve understanding of the molecular sequence of events underlying oocyte maturation.

**Methods:**

Transcriptomic analysis was performed on eight morphologically diverse stages of the ovary, including primary and secondary stages of oocyte growth, ovulation, and atresia. Ovary histology, plasma vitellogenin, 17β-estradiol, and testosterone were also measured to correlate with gene networks.

**Results:**

Global expression patterns revealed dramatic differences across ovarian development, with 552 and 2070 genes being differentially expressed during both ovulation and atresia respectively. Gene set enrichment analysis (GSEA) revealed that early primary stages of oocyte growth involved increases in expression of genes involved in pathways of B-cell and T-cell receptor-mediated signaling cascades and fibronectin regulation. These pathways as well as pathways that included adrenergic receptor signaling, sphingolipid metabolism and natural killer cell activation were down-regulated at ovulation. At atresia, down-regulated pathways included gap junction and actin cytoskeleton regulation, gonadotrope and mast cell activation, and vasopressin receptor signaling and up-regulated pathways included oxidative phosphorylation and reactive oxygen species metabolism. Expression targets for luteinizing hormone signaling were low during vitellogenesis but increased 150% at ovulation. Other networks found to play a significant role in oocyte maturation included those with genes regulated by members of the TGF-beta superfamily (activins, inhibins, bone morphogenic protein 7 and growth differentiation factor 9), neuregulin 1, retinoid X receptor, and nerve growth factor family.

**Conclusions:**

This study offers novel insight into the gene networks underlying vitellogenesis, ovulation and atresia and generates new hypotheses about the cellular pathways regulating oocyte maturation.

## Introduction

Female teleost fishes show remarkable diversity in reproductive strategies. Some reproductive strategies include continuous and semi-synchronous spawning, sex reversal, and synchronous or simultaneous hermaphroditism. Fish that are fractional spawners develop eggs rapidly for fertilization over relatively short time scales (days to weeks) while synchronous spawning fish develop their eggs gradually over an entire breeding cycle (months). Despite the wide diversity in reproductive strategies, there are characteristic morphological and physiological changes that occur as the oocytes grow and mature. In general, active nuclear transcription and DNA recombination drives meiotic divisions of oogonia during primary growth phases of development. The primary oocyte stage is characterized by the formation of the follicle including the granulosa cells, which surround the oocyte, the basal lamina, produced by the granulosa layer and the theca cells including blood vessels. Also, one can discern the beginning of formation of oocyte microvilli, extending towards the granulosa layer, followed by extensions of microvilli from the granulosa layer towards the oocyte. During this phase, meiosis is arrested at the diplotene stage of prophase I and the oocyte is characterized by intensive mRNA transcription [1]. Towards the end of this phase, cortical alveoli are visible in the cytoplasm of the growing oocytes and the network of microvilli extending both from the oocyte and the granulosa towards each other is well formed and there is a distinguishable outer zona radiata layer around the oocyte. Primary oocytes progress into secondary growth phase and are characterized by active uptake of nutritional resources including the egg yolk precursor protein vitellogenin (Vtg) and lipids and active deposition of the zona radiata interna. The significant increase in the rate of Vtg uptake is also associated with a marked increase in cell size. In early stages of oocyte maturation, yolk globules become distinct and visible, eventually fusing into a large, single globular yolk formation that precedes germinal vesicle breakdown and final oocyte maturation and ovulation. In some cases, atresia may occur in which the oocyte is reabsorbed prior to ovulation. Atresia can occur at any stage of oocyte development and this process can be influenced by environmental factors and the individual’s physiological status.

Transcriptomics-based studies in the teleostean ovary have provided valuable insight into the molecular events leading to ovulation. In many cases, the transcriptional response can be associated with the physiological and morphological changes that are occurring in the ovary. Gene expression studies have been performed in teleost fishes with different reproductive strategies, including both fractional and seasonal spawners [2]–[10]. Largemouth bass (LMB) (*Micropterus salmoides*) are widely distributed throughout the southern continental USA. LMB have significant economic value because they are highly prized in the sport-fishing industry, in addition to being ecologically important as apex predators in their freshwater environments. LMB are semi-synchronous reproducers and many populations in central Florida typically spawn during mid to late spring (end of March-early April) when water temperatures are approximately 75°F. Floridian LMB sampled in mid-late spring have higher gonadosomatic indices (GSI) when compared to individuals sampled in other seasons, and in the summer months Florida LMB are sexually recrudescent, exhibiting little ovarian development [11]–[12].

The present study uses a transcriptomics-based approach and bioinformatics to characterize the molecular events in the LMB ovary throughout a complete breeding season. Microarray analysis was conducted for eight distinct histological stages in wild female LMB that included ovulated eggs and ovaries that contained atretic oocytes. We point out here that this study was conducted in the whole ovary and not in individual oocytes. The ovarian stages used to categorize individuals reflect the emerging and dominant oocyte stage within the ovary in relation to the total volume of the ovary. LMB offer the opportunity to characterize gene expression patterns at specific points in the reproductive process, since a cohort of maturing eggs go through the process in a synchronous manner. The present study contributes to an increased mechanistic understanding of the gene networks that are activated and inhibited in the teleostean ovary during oocyte development and identifies potential biomarkers of atresia that could be utilized in both aquaculture and ecotoxicology as predictors of disrupted reproductive capabilities in wild populations of fish.

## Methods

### 2.1 Animals and Experimental Design

Adult female and male LMB were collected by electroshocking from October 2005 to April 2007 at Welaka, FL USA (29.48° N, 81.67° W), located approximately 20 miles south of Palatka, FL on the St. John’s River in an area that is considered to be relatively free from the influence of industrial effluent and agricultural runoff [11]. The period between sampling events ranged from 4–5 weeks and amounted to one sampling event per month. All wild fish were collected in conjunction with colleagues from Florida Fish and Wildlife Conservation Commission and the UF IACUC approved all animal-handling procedures.

Approximately 10 males and 10 females were collected during each sampling period. At the time of capture, 3–5 ml of blood was drawn from the caudal vein using a heparinized vacutainer and stored on ice for Vtg and steroid measurements. Body weight (g), body total length (tip of the mouth to tip of the tail; mm), and gonad weight (±0.01 g) were recorded. Gonadosomatic index (GSI) was calculated for all individuals as [gonad weight/absolute body weight] X 100%. Multiple tissues were harvested at time of dissection for different studies and included brain, liver, ovary or testes, pituitary, and kidney. Tissues were frozen in liquid nitrogen in the field and stored in the laboratory at −80°C until processed for microarray analysis. Ovulated eggs (clear and gold in color), when present in the tissues, were collected directly from an excised ovary by cutting, along the longitudinal plane and flash frozen in liquid nitrogen. All microarrays were performed on ovary tissue except for a set performed on ovulated eggs (OV).

### 2.2. Histology and Staging of LMB Ovaries

Ovaries were fixed in buffered formalin, 1∶20 dilution (Protocol®, Fisher Scientific, MI, USA) and then embedded in plastic and cut into 4 µm sections. Staining of the ovary was done with standard hematoxylin and eosin. Female LMB ovaries were categorized into eight ovarian stages based on histology following the categories described by Grier et al. [13] and used previously by us [12]. The stages included primary growth stages of perinuclear (PN) and cortical alveoli (CA), early vitellogenesis (eVtg), late vitellogenesis (lVtg), early oocyte maturation (eOM), late oocyte maturation (lOM), ovulation (OV), and atresia (AT). The LMB ovary starts out in the fall in the PN state. As environmental temperature decreases, cohorts of oocytes progress through the various stages together, with one cohort leading the way, but followed by additional cohorts of eggs that appear to mature together. Therefore, at any time after the process starts, the ovary is heterogeneous and contains eggs at different stages of development. Ovarian stages of females were classified based on the most prevalent oocyte stage as determined by visual analysis of histological slides at 2×, 10× and 40× magnifications.

### 2.3 Plasma Vitellogenin and Plasma Steroid Levels

Concentrations of plasma Vtg were determined by Enzyme-Linked Immunosorbent Assay (ELISA) using monoclonal antibody, 3G2 (HL1393) as previously described [14]. The limit of detection for the LMB Vtg ELISA was 0.001 mg/mL. All assays were performed in triplicate and reported as the mean of the three measurements. The coefficient of variation was <10% for all samples analyzed. Inter and intra-assay variability were <10%, and <5%, respectively.

Plasma steroids were evaluated after extracting plasma samples with n-butylchloride (Acros Organics) as previously described [15]. Extraction efficiency for each sample was determined by spiking in 65 µL of 50 nCi/mL [^3^H]estradiol or [^3^H]testosterone (Amersham, specific activity of 44 and 73 Ci/mmol, respectively) prior to extraction. Sex steroid levels were determined by ELISA (Immuno-Biological Laboratories, Minneapolis, MN) as previously described [15]. The limit of quantitation for E_2_ and T was 20 pg/mL and 25 pg/mL, respectively. Intraday coefficient of variation was less than 5% across all standards, ranging from 0 to 4.5% as measured for each assay.

### 2.5 Transcriptomic Profiling

Custom LMB oligonucleotide microarrays were designed by the Denslow laboratory [16] and printed in the format of 8×15 K by Agilent (Santa Clara, CA, USA) (GPL13229). Gene annotation was performed by BLAST against the NCBI NR and NT databases. Only well-annotated transcripts (NR e value<10^−4^) were used for pathway analyses.

Gene expression analysis was performed with four biological replicates for each ovarian stage (N = 32 microarrays). Total RNA for microarray analysis was extracted from LMB ovary samples using RNA STAT-60 reagent (TEL-TEST Inc., Friendswood, TX, USA). RNA purity and quantity were measured using the NanoDrop ND-1000 (Nanodrop Technologies, Wilmington, DE) and RNA integrity was evaluated using the Agilent 2100 BioAnalyzer (RNA 6000 Nanochip) and had average RNA integrity values ± SD of 8.9±0.48; RINs>8). Microarray hybridizations were performed according to the Agilent One-Color Microarray-Based Gene Expression Analysis protocol using Cyanine 3 (Cy3). Microarrays were scanned at 5 µm at both 10 and 100 PMT (Agilent G2505 B Microarray Scanner), as previously described [17]. All raw microarray data for this experiment have been deposited into the NCBI Gene Expression Omnibus (GEO) database (series GSE32930; platform GPL13229).

### 2.6 Bioinformatics

Raw expression data were analyzed by two different methods. *Method 1:* ANOVA using JMP® Genomics v4.0 software. Raw intensity data for each microarray was normalized using LOESS normalization with a smoothing factor of 0.2 or by Quantile normalization and the two methods were in close agreement in identifying differentially expressed transcripts. The two normalization procedures identified >90% of the same transcripts as differentially expressed and a regression of fold-change estimates between the two normalization methods for transcript fold change was R^2^>0.95. One microarray slide in the atresia group had a low global intensity signal relative to all other slides and was removed from all downstream analyses. Differentially expressed transcripts from one stage to the next were identified using a one-way analysis of variance (ANOVA) followed by an FDR (5%) post hoc test for multiple comparisons.

*Method 2: Significance analysis of microarrays (SAM*). To identify genes which may change as a result of a linear sequential development we performed a SAM [18] time course analysis. Here each developmental sequence is summarized by a slope and genes are identified through a standard one class SAM analysis. Genes identified by this approach will show a gradual increase or decrease in their expression over the different developmental stages. Because this approach does not generate fold changes, the fold change was measured as the difference between the highest and the lowest expression value over all ovarian stages. In order to simplify the analysis, we assumed that oocyte development would progress in a linear fashion towards ovulation or towards atresia. Therefore, there were two analyses that included the following trajectories based upon two different scenarios for a maturing oocyte: (A) PN -> CA -> eVtg -> lVtg -> eOM -> lOM -> OV and (B) PN -> CA -> eVtg -> lVtg -> eOM -> lOM -> AT. SAM identifies those transcripts that are most likely to significantly change over time. Attention was given to receptor mRNAs in the ovary to identify putative signaling pathways involved in oocyte development and maturation.

Hierarchical clustering was performed in JMP Genomics, using the Fast Ward algorithm. For some analyses, data sets for primary growth stages (PG and CA), vitellogenesis (eVtg and lVtg), and maturation (eOM and lOM) were combined to strengthen the power of the bioinformatics analysis. OV and AT remained separate. Functional enrichment was conducted in JMP Genomics and an adaptive false discovery rate was applied with 10,000 bootstrap iterations.

PathwayStudio™ 7.1 (Ariadne, Rockville, MD, USA) was used for GSEA. LMB genes were annotated with human homologs using Entrez Gene IDs for pathway enrichment (using RefSeq database and e values<10^−4^). For GSEA, genes were permutated 400 times using the Kolmogorov-Smirnov classic approach as an enrichment algorithm. To broaden the analysis, all pathways were expanded to include cell processes and functional classes (enrichment p<0.05). GSEA was conducted on curated pathways in Pathway Studios. A sub-network enrichment analysis (SNEA) was performed to determine which gene expression networks were differentially activated/inhibited during follicular development.

Described by Subramanian et al. [19], GSEA uses defined gene sets that are based on known biological knowledge, for example gene ontology. Transcripts are ranked as a list based on p-value for example and a Kolmogorov-Smirnov statistical comparison is conducted to determine if a given gene ontology is found more often at the top of the list (i.e. enriched) versus being randomly distributed throughout the list. In contrast, SNEA builds gene networks based on expression relationships or common cell functions among genes. SNEA builds sub-networks beginning with an entity (e.g. gene or protein) that is common among all other entities. An algorithm calculates a background distribution of expression in the gene list. This is followed by a statistical comparison between the sub-network and the background distribution of the gene list obtained from largemouth bass ovary using a Mann-Whitney U-Test. A p-value is generated that indicates the statistical significance of difference between two distributions. This approach has been applied successfully to biomarker discovery in mammals [20] and for gene and protein networks in teleost fishes [21], [22].

### 2.7 Real-Time PCR

Real-time PCR assays for membrane progestin receptor alpha (mPR-α,) growth hormone receptor 1(ghr-1), luteinizing hormone receptor (lhr), and follicle-stimulating hormone receptor (fshr) were developed because of their key roles in oocyte development. Transcripts for mPR-α and ghr were present on the microarray. Total RNA used for real-time PCR was DNase treated with DNAse TURBO (Ambion, Austin, Texas, USA) as per manufacturer’s protocol. cDNA synthesis using Superscript-II was performed using 3 µg of RNA (Invitrogen, Carlsbad, CA, USA). Detailed methods for cloning and sequencing LMB genes have been previously described [12]. Primers of 18–22 base pairs (bp) with optimal annealing temperature ∼60°C were designed using Primer 3 [23] to amplify sequences between 137–299 bp (Table S1 in File S2). Primers for real-time PCR were initially tested using LMB ovary cDNA and the amplicons were sequenced to verify specificity [12].

Standard curves were used to obtain copy numbers of each gene and ranged from 1×10^8^ to 1×10^1^ copy number. Standard curves showed between 90–110% efficiency and were linear at R^2^>0.990. Two minus RT controls from separate cDNA sample pools and four no template controls (water) were run on each plate to ensure genomic DNA contamination was negligible and not a factor after DNase treatment. Melt curves were also generated for all genes on every real-time PCR plate to verify a single product/dissociation curve.

Real-time PCR analysis of gene expression was performed using 1X iQ SYBR Green Supermix (Bio-Rad, Hercules, CA) as previously described (Martyniuk et al., 2009). Sample sizes for real-time PCR assays were as follows; PN (n = 9), CA (n = 8), eVtg (n = 8), lVtg (n = 3), OM (n = 12) and AT (n = 2). Total number of animals used in the stage specific analysis was n = 42. During analysis of qPCR data, significant variation in *18S rRNA* expression by month and by stage (p<0.001) was noted in ovarian samples. Trends in expression of conventional housekeeping genes by reproductive stage in ovarian tissue from aquatic species have been previously reported in the literature [15], [24], [25] and methodologies have been developed to standardize housekeeping gene expression values when such a pattern of variation is found [26], [27]. Therefore, *18S rRNA* data were normalized using the method utilized in the most recent publication on LMB ovarian gene expression [15]. Standardized *18S rRNA* values were then used to normalize the gene of interest (GOI) as a simple ratio of GOI input amount/standardized *18S rRNA* for each individual sample.

### 2.8 Statistics

All physiological endpoints and gene expression patterns were assessed for normality using a Shapiro-Wilk W test and homogeneity of variance using a Levene’s test. Data were tested for normality after log_10_ transformation. One-way analysis of variance (ANOVA) followed by a Dunnett’s T3 post-hoc test for multiple comparisons were conducted on data that met assumptions of normality and homogeneity. Wilcoxon/Kruskal-Wallis Tests (Rank Sums) were used if data did not meet assumptions. Differences were considered statistically significant when p<0.05. All analyses were performed in JMP Genomics (V4.0).

## Results

### 3.1 LMB Ovarian Histology

**Figure 1:**
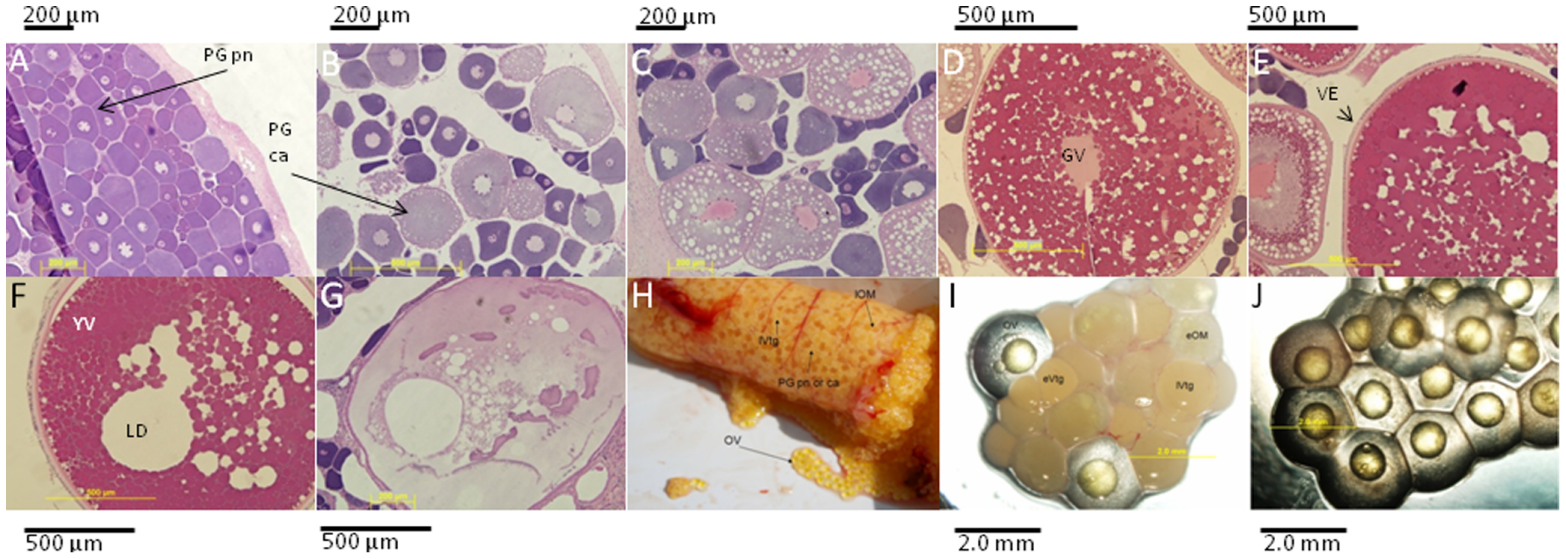
Representative ovarian stages used to characterize gonad phase. (A) primary growth perinuclear (PG pn), (B) primary growth cortical alveoli (PG ca) (C) secondary growth early vitellogenesis (SG eVtg), (D) secondary growth late vitellogenesis (SG lVtg) (E) early oocyte maturation (SG eOM), (F) late oocyte maturation (lOM) (G) Atresia (AT) (H) external morphology of the heterogeneous LMB ovary, (I) oocytes at various stages of development, and (J) ovulated eggs that have not been water hardened. Additional abbreviations are as follows: Germinal Vesicle (GV), Lipid Droplet (LD), Vitelline Envelope (VE), and Yolk Vesicles (YV). Scale bars correspond to 200 µm (A–B) and 500 µm (C–F).

Figure 1 shows representative micrographs for each of the LMB ovarian stages. The PN stage is characterized as having perinuclei in the absence of gametogenesis (A). The CA stage is characterized as a differentiated ovary containing cortical alveoli and non-vitellogenic oocytes (B). Early Vtg is characterized as the initial uptake of Vtg into oocytes (increasing acidic pH within the oocyte) (C), while late Vtg is marked by significant Vtg deposition surrounded by the formation of multiple oil droplets (D). Early OM oocytes also contain multiple oil droplets (E), while late OM oocytes are characterized by the presence of one large coalesced oil droplet (F). There was a dramatic loss of staining color for atretic oocytes and visual evidence of GVBD (G). Ovulated eggs (J) were collected into pans.

The fact that not all of the oocytes mature exactly at the same time in LMB ovary results in partial overlap in molecular signatures between closely related stages (e.g. early maturation versus late maturation). But, the stages were incremental, with each new stage characterized by visual changes in the overall appearance of the histological section.

### 3.2 GSI, Vtg, and Sex Steroids in Wild Female LMB

**Figure 2:**
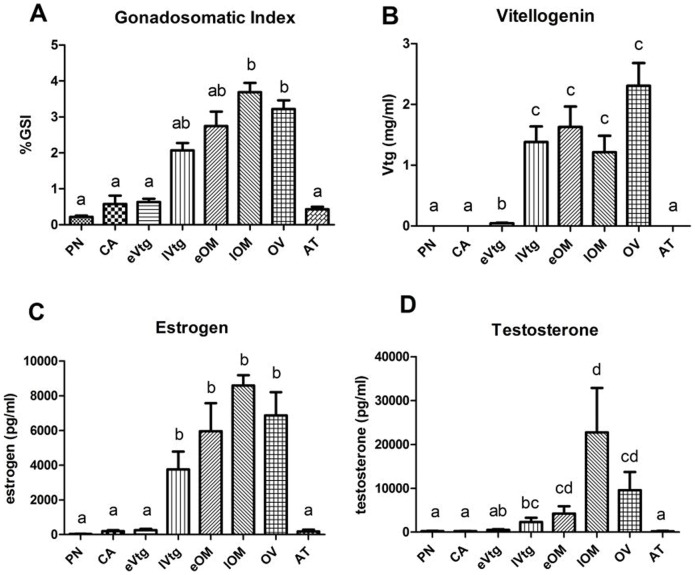
Reproductive endpoints in female largemouth bass. (A) Gonadosomatic index (GSI), (B) vitellogenin (Vtg), (C) 17β-estradiol (E_2_) and (D) testosterone (T) are shown at each stage for all individuals that were used in the microarray analysis (n = 4/stage). Data are presented as mean (±SEM) and were analyzed using a one-way ANOVA (log transformed data for Vtg and T) followed by a Dunnett’s T3 post-hoc test. Different letters denote significant differences among groups (p<0.05).

Log transformation of T had a normal distribution and was evaluated with ANOVA while GSI, Vtg, and E_2_ were not normally distributed and were evaluated with Wilcoxon/Kruskal-Wallis. There was significant variation in GSI, Vtg, E2, and T across stages (Figure 2). GSI ranged in individual females from 0.07% (CA) to 4.39% (lOM) (Figure 2A) and significantly increased throughout ovarian development (d.f. = 7; *F* = 27.8; p<0.001), reaching a peak at lOM and OV. Plasma Vtg ranged in individual females from below detection (PG) to 2.91 (mg/ml) (OV) (Figure 2B) and significantly varied with stage (d.f. = 7; *F = *28.3; p<0.001) peaking during lVtg. Plasma Vtg remained elevated and constant in later stages of oocyte growth (OM) and dramatically decreased in animals exhibiting AT.

E_2_ ranged in individual females from 3.54 (pg/ml) (PG) to 10695.89 (pg/ml) (OV) (Figure 2C). E_2_ in females significantly increased throughout reproductive maturation (d.f. = 7; *F* = 26.5; p<0.001) and followed closely the changes in GSI, peaking at late secondary growth phases and remaining relatively constant until AT. T ranged in individual females from 83.34 (pg/ml) (AT) to 52235.71 (pg/ml) (lOM) (Figure 2D). T in females also significantly increased throughout follicular maturation (d.f. = 7, *F = *21.60, p<0.001), peaking during late secondary growth phases and remaining relatively constant until AT. Plasma T in LMB are consistent with other reports in laboratory and wild bass [28], [29]. Lastly, Pearson correlations were performed on each reproductive endpoint (GSI, Vtg, and steroids) and all variables were significantly and positively correlated (p<0.01). Pearson correlation coefficients ranged between 0.491 for Vtg and T to 0.869 for GSI and E_2_.

### 3.3 Ovarian Stage-Specific Transcriptomics

The cluster analysis for all transcripts on the LMB oligonucleotide microarray demonstrated that gene expression patterns for each sequential ovarian phase was most similar to the prior stage (Figure S1 in File S1), suggesting that gene expression profiles were related to ovary morphology. Primary growth stages (PN and CA) formed a clade and so did secondary growth stages (Vtg and OM). Expression profiles for atresia were most different when compared to all the other ovarian stages.

The marked differences in gene expression clustering were also reflected in the number of transcripts that were differentially expressed from stage to stage. Using the criteria of FDR adjusted p-values (5%), NR e-value<10^−4^, and fold change>±1.5, the number of differentially expressed probes for each stage were as follows; PN to CA (n = 0), CA to eVtg (n = 0), eVtg to lVtg (n = 9), lVtg to eOM (n = 351), eOM to lOM (n = 0), lOM to OV (n = 94) and lOM to AT (n = 168). Therefore, gene expression changes were more pronounced with major transitions in ovary morphology.

When closely related stages were grouped together to increase power of the analysis and using the same criteria as above, the following numbers of genes were differentially expressed; Primary growth (PN+CA) to secondary growth (eVtg+lVtg) (n = 645), secondary growth to maturation (eOM+lOM) (n = 397), and maturation to ovulation (OV) (n = 552) and maturation to atresia (AT) (n = 2070). A complete list of these genes is provided in File S3.

### 3.4 Transcripts Significantly Altered Over Time in the Ovary

**Figure 3:**
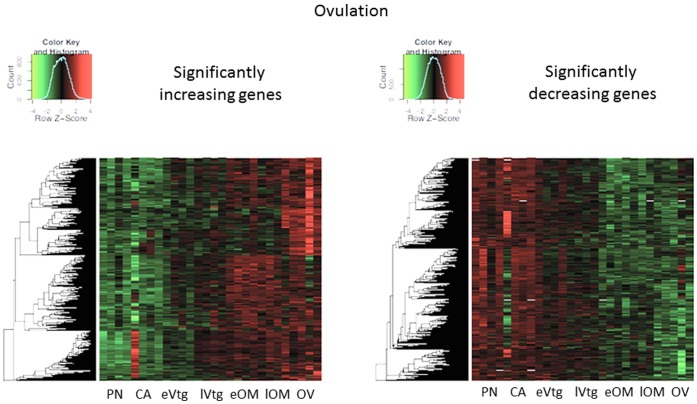
Heat maps for genes that showed significant increases and decreases in mRNA abundance at ovulation after a time course analysis (SAM).

**Figure 4:**
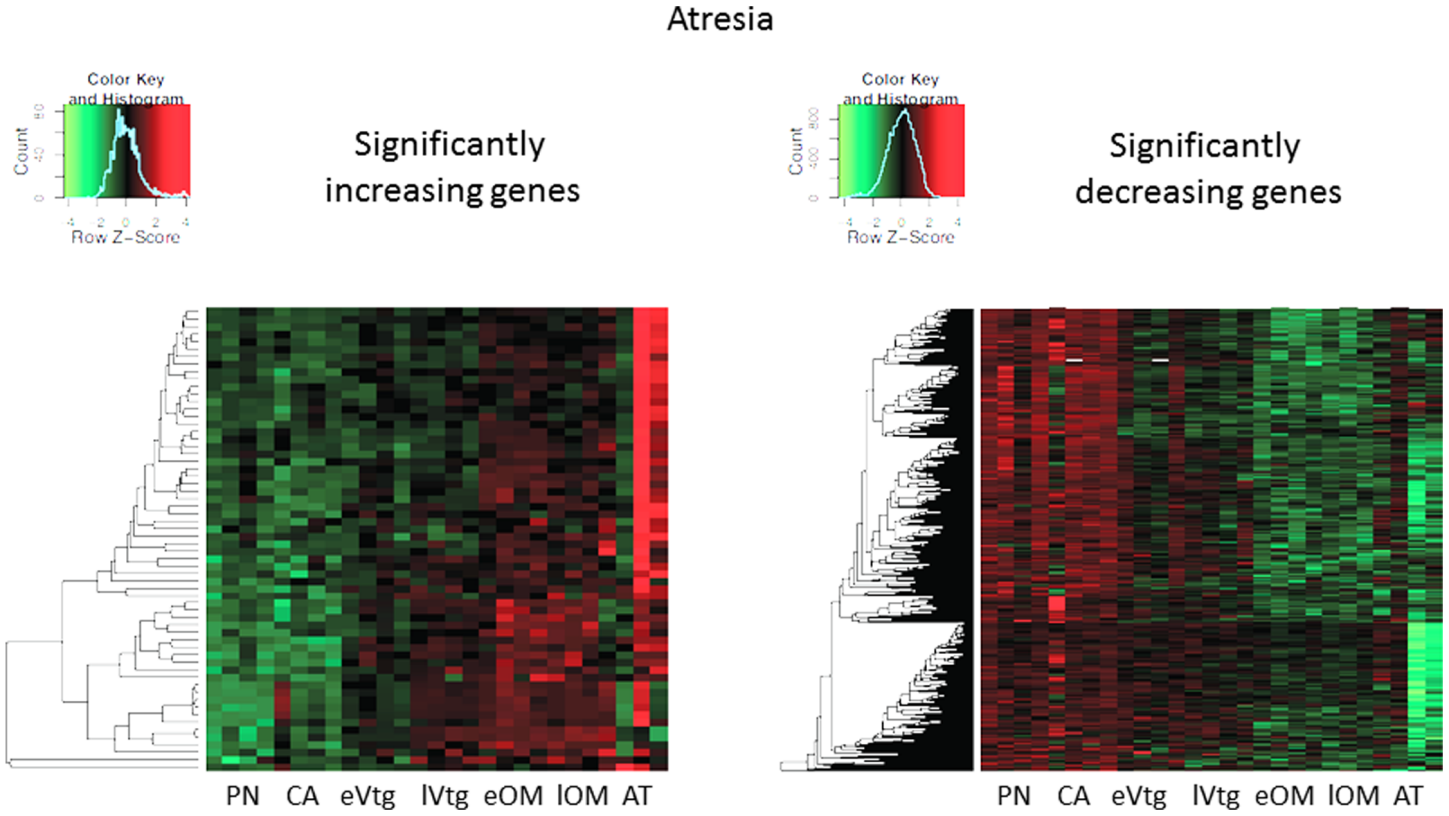
Heat maps for genes that showed significant increases and decreases in mRNA abundance at atresia after a time course analysis (SAM).

SAM identified 1020 transcripts that increased with oocyte development and 1444 transcripts that decreased with abundance in the trajectory towards ovulation (PN -> CA -> eVtg -> lVtg -> eOM -> lOM -> OV), whereas 62 genes were significantly increased and 599 genes decreased in progression towards AT (PN -> CA -> eVtg -> lVtg -> eOM -> lOM -> AT). All significant genes are plotted on separate heat maps showing ovulation (Figure 3) and atresia (Figure 4).

Within the genes that were identified by the SAM algorithm, gene titles were searched for ‘transcription’ or ‘receptor’ (FDR corrected at 5%). There were 17 genes that fit this search that increased in abundance (Figure S2A in File S1) and 10 genes that decreased (Figure S2B in File S1) within the OV analysis. There were 15 genes that were significantly decreased within the AT analysis (Figure S2C in File S1). All differentially expressed transcripts involved in receptor signalling as well as transcription factors identified by the SAM are provided in Table S2 in File S2.

### 3.5 Bioinformatics: Functional Enrichment, GSEA, and SNEA

Biological processes significantly over-represented (over) or under-represented (under) in each ovarian stage are listed in Table S3 in File S2. A gene was assigned a binary category value of 1 if it passed the FDR correction and 0 if it did not pass the FDR. All data (BP, MF, and CC) from the functional enrichment analysis are provided in File S4.

Some noteworthy examples of biological processes associated with the transition from PG to SG were glycolysis, ion transport, and chloride transport. For the transition from SG to OM, lipoprotein metabolic processes, electron transport, chloride transport, ubiquinone biosynthetic process, and aromatic compound metabolic processes were affected and for the transition from OM to OV, lipid transport, chloride transport, and cellular iron ion homeostasis were affected. Some noteworthy examples of biological processes affected with atresia were blood coagulation, lipid transport, protein import into mitochondrial inner membrane, notch signaling pathway, and cell proliferation.

**Figure 5:**
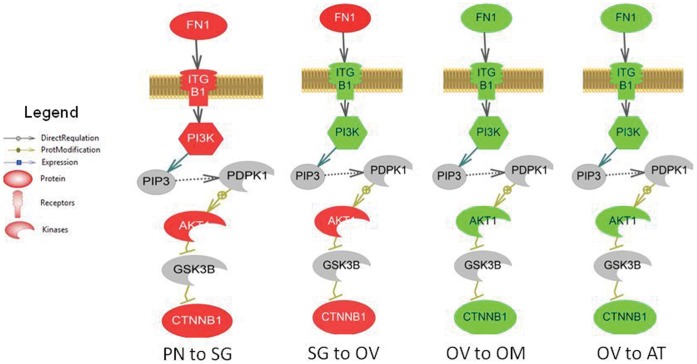
Fibronectin receptor - catenin (cadherin-associated protein), beta 1 (CTNNB) signalling pathway during oocyte development. This pathway was significantly increased at early growth stages (red) and is decreased at ovulation and atresia (green). The abbreviations are provided in the supplementary abbreviation list (File S5).

Gene set enrichment results for ovarian stages are shown in Table S4 in File S2. Major pathways that were increased in expression at early stages of follicular growth were Notch signaling, T-cell and B-cell receptor activation, and fibronectin, epidermal growth factor, and adenosine receptor signaling. The NK cell activation pathway is also significantly up-regulated ∼15–20% at early stages of ovarian development. At ovulation, many of these pathways were down-regulated. Pathways depressed at ovulation also included adrenergic receptor signaling, sphingolipid metabolism, and natural killer cell activation. At atresia, cell signaling pathways that are involved in gap junction and actin cytoskeleton regulation, gonadotrope and mast cell activation, and vasopressin receptor signaling were down-regulated while oxidative phosphorylation pathways and reactive oxygen species metabolism were increased. The largest difference in the gene networks were that atresia was marked with significant changes in cell structure relative to the other stages. In contrast to early growth stages, the NK cell activation pathway is significantly depressed by approximately 30% during atresia. Figure 5 shows an example of a curated pathway, and shows changes in members of the fibronectin receptor - catenin (cadherin-associated protein), beta 1 (CTNNB) signalling pathway over follicular development. This pathway was significantly increased at early follicular growth stages (red) and is decreased at ovulation and atresia (green).

SNEA identified a number of expression networks that were involved in the different stages of follicular development (Table 1). Some examples include expression targets for LH signaling, which were depressed during vitellogenesis but increased 150% at ovulation. Other networks found to play a significant role in oocyte maturation included genes regulated by activins and inhibins, neuregulin 1, retinoid X receptor, alpha, nerve growth factor family, STAT5A, bone morphogenic protein 7, and toll-like receptor 4.

**Table 1:** Sub-network enrichment analysis of expression targets altered during the major transitions of ovarian development.

Stage Transition	Gene Network Expression Seed	Total # of Neighbors	# of Measured Neighbors	Median change	p-value
PG to SG	corticotropin releasing hormone	54	10	−1.541	0.0359
PG to SG	adenylate cyclase	57	10	−1.409	0.0358
PG to SG	activin	89	20	−1.409	0.0177
PG to SG	neuregulin 1	87	23	−1.357	0.0192
PG to SG	basic-helix-loop-helix protein	71	14	−1.228	0.0021
PG to SG	retinoid X receptor, alpha	115	27	1.252	0.0164
PG to SG	parathyroid hormone 1 receptor	107	16	1.304	0.0383
PG to SG	nuclear receptor co-repressor 1	43	10	1.405	0.0097
PG to SG	kininogen 1	51	12	1.405	0.0019
PG to SG	nuclear factor I/C	56	14	1.405	0.0251
SG to OM	luteinizing hormone	51	12	−1.570	0.0023
SG to OM	transcription factor AP-2 alpha (activating enhancer binding protein2 alpha)	90	16	−1.453	0.0131
SG to OM	upstream transcription factor 2, c-fos interacting	65	15	−1.237	0.0488
SG to OM	kininogen 1	51	12	1.243	0.0242
SG to OM	fibronectin 1	83	11	1.279	0.0328
SG to OM	nuclear factor I/C	56	14	1.285	0.0037
SG to OM	nuclear receptor co-repressor 1	43	10	1.413	0.0009
OM to OV	nuclear receptor subfamily 1, group H, member 4	100	21	1.212	0.0304
OM to OV	leptin	243	39	1.237	0.0390
OM to OV	calcium channel	41	13	1.376	0.0078
OM to OV	nuclear receptor subfamily 5, group A, member 1	53	17	1.451	0.0327
OM to OV	adenylate cyclase activating polypeptide 1	100	26	1.578	0.0251
OM to OV	retinoic acid receptor	52	11	1.719	0.0082
OM to OV	forkhead box A2	71	15	1.719	0.0336
OM to OV	ghrelin/obestatin prepropeptide	55	11	1.744	0.0118
OM to OV	nuclear receptor subfamily 5, group A, member 2	49	10	1.798	0.0201
OM to OV	basic-helix-loop-helix protein	71	14	1.945	0.0008
OM to OV	calmodulin-dependent protein kinase	53	13	1.955	0.0092
OM to OV	activin	89	20	1.955	0.0059
OM to OV	corticotropin releasing hormone	54	10	2.100	0.0051
OM to OV	nerve growth factor family	40	10	2.100	0.0073
OM to OV	bone morphogenetic protein 7	82	14	2.100	0.0086
OM to OV	adenylate cyclase	57	10	2.438	0.0013
OM to OV	luteinizing hormone	51	12	2.438	0.0015
OM to AT	nuclear receptor co-repressor 1	43	10	−6.023	0.0037
OM to AT	signal transducer and activator of transcription 5B	49	10	−2.400	0.0070
OM to AT	fibronectin 1	83	11	−2.400	0.0166
OM to AT	inhibin, beta A	109	27	−2.364	0.0030
OM to AT	toll-like receptor 4	114	12	−2.310	0.0408
OM to AT	retinoid X receptor, alpha	115	27	−2.245	0.0211
OM to AT	nuclear receptor subfamily 2, group F, member 2	43	11	−2.032	0.0379
OM to AT	kininogen 1	51	12	−1.576	0.0162
OM to AT	gonadotropin	157	34	−1.554	0.0085
OM to AT	forkhead box A2	71	15	1.468	0.0454

Total number of neighbours are those known genes/proteins that are affected by the gene set seed and measured number of neighbours are those genes that are present on the LMB microarray. Median fold change indicates the overall change in the group. Listed here are those gene networks that have changed more than 20% and have more than 10 measured neighbours in the network. All significantly affected networks in the ovary are provided in File S4.

**Figure 6:**
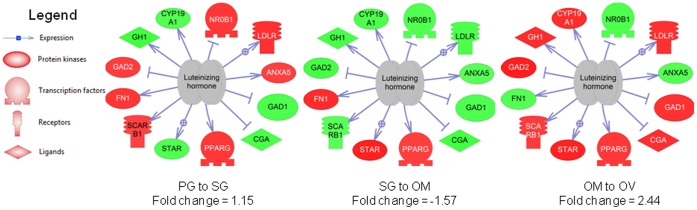
Expression targets for luteinizing hormone, a well described hormone involved in ovulation. These targets were significantly increased at both early stages and ovulation, but decreased during vitellogenesis. Red indicates that the gene is increased and green indicates that the gene is decreased. The abbreviations are provided in the supplementary abbreviation list (File S5).

**Figure 7:**
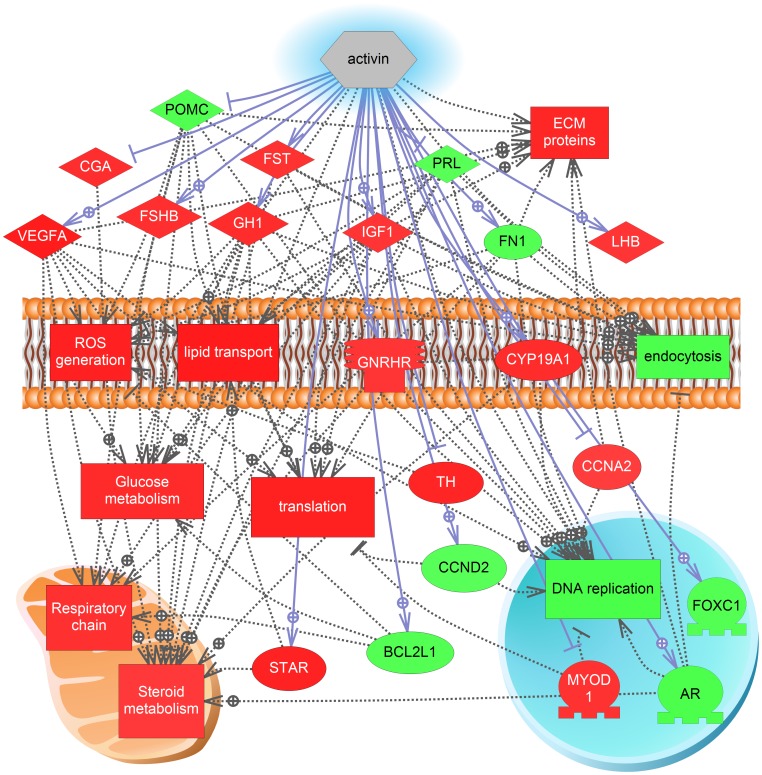
Activin expression network at ovulation. Significantly affected cell processes are mapped onto the gene network. Red indicates that the gene is increased and green indicates that the gene is decreased. The abbreviations are provided in the supplementary abbreviation list (File S5).

**Figure 8:**
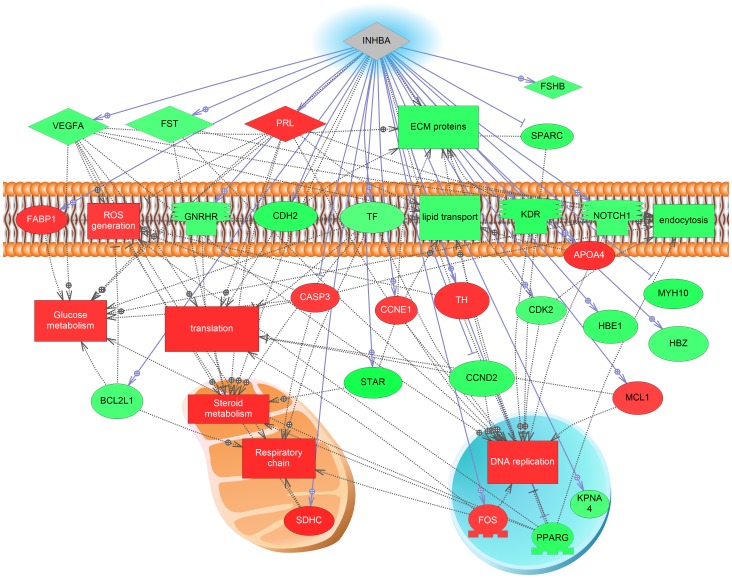
Inhibin expression network at atresia. Significantly affected cell processes are mapped onto the gene network. Red indicates that the gene is increased and green indicates that the gene is decreased. The abbreviations are provided in the supplementary abbreviation list. The network was similar in the effects on energy production and steroid metabolism but differed in extracellular matrix proteins, lipid transport, and DNA replication compared to the activin network. The abbreviations are provided in the supplementary abbreviation list (File S5).

We provide some examples of the SNEA analysis. Expression targets of luteinizing hormone, a hormone well known to be involved in the final stages of ovulation, are low during vitellogenesis but increased over 150% at OV (Figure 6). This analysis identifies downstream targets of LH signaling that may be important for ovulation. Two interesting differences between ovulation and atresia were the expression networks for activin and inhibin. A gene network for activin was significantly increased 96% at ovulation (Figure 7) while a gene network for inhibin B was significantly decreased 136% during atresia (Figure 8). As with the GSEA analysis, red indicates an increase in expression while green indicates a decrease in expression levels. The major cell processes that were significantly associated with the genes involved in the network were also included (connectivity <5) and represent many of the cell signaling cascades identified by the GSEA. For the cell processes that were mapped to the two gene expression networks, the processes of DNA replication, lipid transport, and extracellular protein complexes were differentially impacted between ovulation and atresia. Energy storage and metabolism were increased in both networks. In the inhibin B network, cell respiration and reactive oxygen species were increased while extracellular protein networks were depressed in the trajectory to atresia, which corresponds to the GSEA analysis.

### 3.7 Ovarian Stage-Dependent Expression of mPR-α, and ghr1, lhr,and fshr mRNA

**Figure 9:**
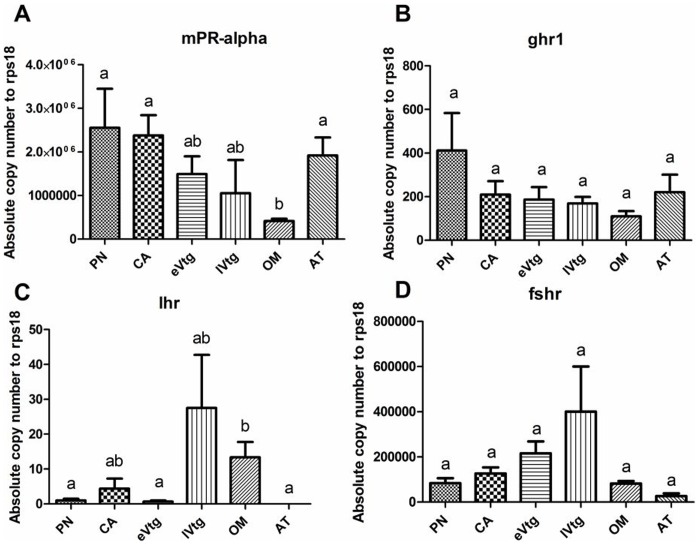
Ovarian stage dependent expression of genes involved in reproduction. Genes are (A) *mPR-alpha* (B) *ghr1* (C) *lhr* and (D) *fshr*. Expression is reported as mean absolute copy number of the transcript ± SEM. Abbreviations for female phases are as follows; Primary growth perinuclear (PG pn); Primary growth *cortical alveoli* (PG ca); Secondary growth early vitellogenesis (eVtg); Secondary growth late vitellogenesis (lVtg); Oocyte maturation (OM); and Atresia (AT). Sample sizes/month is n = 4 except for the month of June (n = 3). PG pn (n = 9), PG ca (n = 8), eVtg (n = 8), lVtg (n = 3), OM (n = 12), and AT (n = 2). Total number of animals used in the stage specific analysis was n = 42. Different letters indicate statistical differences among groups (p<0.05).

Gene expression data were log_10_ transformed and data were determined to be normally distributed. Data were grouped according to month (Figure S3 in File S1) and ovarian stage (Figure 9) There was significant variation in the mRNA expression of *mPR-alpha* in the female ovary by both month (d.f. = 11, F = 5.65, p<0.001) and stage (d.f. = 5, F = 7.10, p<0.001). *mPR-alpha* showed higher expression during the primary growth stages (PN and CA) compared to OM. There were no significant variations in *ghr1* expression based on either month (d.f. = 11; F = 1.78, p = 0.098) or stage (d.f. = 5, F = 0.84, p = 0.55).

There was significant variation in the ovarian mRNA steady state abundance of *lhr* by month (d.f. = 11, *F* = 3.42, p = 0.003) and by stage (d.f. = 5, *F* = 5.39, p<0.001). There was also significant variation in *fshr* mRNA levels by month (d.f. = 11; F = 7.78, p<0.001), while differences in *fshr* expression between ovarian stages were not significant. As reported in other teleost fishes, LMB *fshr* showed a different pattern of expression compared to *lhr* and was significantly higher prior to OV while *lhr* mRNA levels were higher during OV.

The expression patterns of *mPR-alpha* and *ghr1* were corroborated by both real-time and microarray data in this study, and each approach revealed that there were higher levels of the transcripts at earlier stages of ovary development. The transcripts *lhr* and *fshr* were not present on the microarray but were examined with real-time PCR. Transcript expression patterns for genes such as *star* and *esrba* have also been previously described in the LMB ovary [30]–[32] and corroborate the ovarian transcriptomics data presented here. For example, the microarray data suggest that *star* is increased in expression towards oocyte maturation and that *esrba* is also increased in expression at OV and at AT.

## Discussion

### 4.1 Ovarian Stage-Specific Transcriptomics

Gene expression patterns of many genes in the maturing LMB ovary (e.g. protocadherins, cathepsins, apolipoproteins, immune-related, steroid-related, and ribosomal genes) corroborate other reports in fish; rainbow trout [4], [6], [33], fathead minnow (*Pimephales promelas*) [9], zebrafish (*Danio rerio*) [34], and Senegalese sole [8]. Thus, there are conserved molecular events that correspond to maturation and development of oocytes within the teleostean ovary. Seasonal variation in physiological endpoints (GSI, plasma Vtg, sex steroids) in LMB is also consistent with reports in other female bass species such as European and striped bass [25], [35]. In the following discussion we highlight some of the major genes and pathways involved in oocyte growth, ovulation, and atresia.

### 4.2 Primary Growth Stages and Transition to Secondary Growth

Oocytes in the PN stage begin to accumulate oil droplets, which marks early transition into pre-vitellogenesis. This transition is predominantly dependent upon water temperature and photoperiod; for LMB populations located in Florida, this begins in early to mid-Autumn. Secondary growth is characterized by the active accumulation of plasma Vtg as well as other nutrients important for growth of the oocytes. Vitellogenesis is marked by active receptor-mediated endocytosis of Vtg and other very low density lipoproteins. Related to these events, notable transcripts with higher expression in secondary growth phases included genes involved in mitochondrial function (*cytochrome b* and *c*, *NADH dehydrogenase subunit 5).* Other regulated genes included *cytochrome P450 1A, cholecystokinin, low density lipoprotein receptor (ldlr),* and apolipoproteins while some notable transcripts with lower expression during vitellogenesis compared to primary growth stages included *follistatin* and *growth hormone receptor 1*.

The growth of oocytes is associated with increased mitochondrial activity and ATP production in preparation for active uptake of lipids and nutrients. Thus, the increase in mRNA levels of genes involved in mitochondrial respiration is expected. Transcript levels of *cytb* in LMB were increased approximately 10-fold progressing towards vitellogenesis, and later decreased at late stages of maturation. Previous studies have also documented increases in *cytb* expression in freshwater anguilliformes [36] as well as *cytb*, *cytc* and NADH dehydrogenase subunits in Senegalese sole [8]. In sole, the processes of mitochondrial/cell metabolism and oxidation-reduction were involved in vitellogenesis. These molecular events coincide with the increase in metabolic activity accompanying the uptake of nutrients during vitellogenesis.

In the LMB ovary, the significant increase in *ldlr* mRNA corresponds to active uptake of VLDL into the oocytes at early stages of vitellogenesis. The induction of lipid-transport genes during oocyte growth has been demonstrated in numerous studies. Agulleiro et al. [37] investigated the expression of *vldr* mRNA in ovarian sections of Senegalese sole during pre-vitellogenesis and reported a positive correlation between *vldlr* transcript levels and the percentage of pre-vitellogenic oocytes. The authors go on to suggest that transcript levels may be used as a precocious functional molecular marker of the number of oocytes recruited for vitellogenesis. In a recently published study, LMB vitellogenin receptor (*vtgr*) mRNA was most highly expressed during PN and CA, just before the VTGR protein is required on the plasma membrane for VTG endocytosis into oocytes [38]. In addition, there was an increase in apolipoprotein expression throughout LMB oocyte development, including *preproapolipoprotein A-I*, *apolipoprotein A-IV4 (apoA-IV4*) and *apoe,* but the expression was higher for some (7 to 8-fold) during the secondary growth phase. Apolipoproteins function in the transport of cholesterol, the precursor for all steroid hormones. Other studies have also seen a similar increases in apolipoprotein mRNA. For example, in coho salmon, *apoe* is significantly higher during early secondary growth stages compared to early growth stages [5]. Therefore, studies report increases in gene related to metabolism, and fatty acid mobilization and transport that are related to Vtg uptake.

### 4.3 Differentially Expressed Transcripts: Ovulation

Ovulation involves the rupture of the cell wall and oocyte expulsion. It has been reported that inflammatory responses are associated with ovulation as well as protease activity, vasodilation, and leukocyte activation [2]–[4]. Transcripts showing a high abundance at ovulation included *star*, apolipoproteins, *glutamate decarboxylase 2*, *claudin 19, steroidogenic factor 1 (sf1)*, *cytochrome p450 3A68*, *growth hormone receptor 1 (ghr1)*, *aromatase (cyp19a)*, *ldlr*, *neurogranin*, *selenoprotein P*, and fibrinogens. Transcripts showing a lower abundance at ovulation included *protocadherin b*, *progranulin, aquaporin 1, cathepsin D*, *cyclin G2,* and *serine proteinase inhibitor*. Many of the LMB transcripts identified here have been previously identified as differentially expressed in specific ovarian stages [4], [5], [39]. In this study, ovarian *star* transcript levels were significantly and positively correlated with plasma E_2_ levels in LMB during various stages of the female reproductive cycle, peaking during maturation and significantly decreasing post-maturation in atretic eggs. In previous studies, we have measured LMB *star* mRNA during late vitellogenesis and maturation and these data correspond to the microarray data [32].

SAM identified a number of transcription factors and receptor subunits that showed significant temporal changes during follicular development that may be important for final ovulation. Transcripts for *esrba*, *lldr*, *progestin and adipoQ receptor family member VII* mRNA levels decreased towards ovulation. These data are consistent with previous reports that *esrba* mRNA in the LMB ovary decreases throughout the stages of LMB ovarian development towards ovulation [31].

Interestingly, during the progression of primary growth stages to ovulation, gamma-aminobutyric acid (GABA) receptor subunits (*gabrb2*, *gabrb3*, *gabrg2*) and peroxisome proliferator-activated receptor gamma (*pparg)* were significantly increased in abundance. Glutamate decarboxylase 67 (g*ad1)*, the enzyme that decarboxylates glutamate to form GABA, was also differentially expressed in early transitions of the ovary and was expressed at higher levels during earlier stages of development. GABA_A_ receptors are ionic Cl^-^ channels that are composed of five subunits and the subunit composition of the GABA_A_ receptor determines the mechanistic properties of the receptor. The role of GABA in the control of reproduction in teleost fishes is well established from a neuroendocrine viewpoint [40], but its role in teleostean ovarian function is not well understood. Although classically defined as a neurotransmitter, GABA and its receptors are found in relatively high abundance in the human ovary and other mammalian reproductive tissues such as the uterus [41]. GABA’s role in teleostean ovarian function has not been studied and the gene expression data presented in this study offers a new hypothesis regarding a potential role for GABA signaling in regulation of oocyte development.

### 4.4 Differentially Expressed Transcripts: Atresia

Prior to ovulation, oocytes not needed undergo atresia, which is characterized by hyperplasia of the oocyte, breakdown of cell structures, and reabsorption of the zona pellucida. Interestingly, during this transition, our data indicates that there is preponderance of down-regulated transcripts, similar to data reported for the Senagalese sole [8]. Transcripts for GABA receptor subunits, *star, ovulatory protein-2*, *putative senescence-associated protein, Na-K-Cl cotransporter, Williams syndrome transcription factor* and *thyroid hormone receptor interactor 11*, among others were down-regulated 10–70 fold. Many of the genes involved with mitochondrial respiration (ATPase subunits, NADH subunits, and cytochrome genes) were also decreased, perhaps reflecting a lowered requirement for energy stores.

Garczynski and Goetz [42] previously identified *ovulatory protein-2* as important for ovulation and it was significantly depressed in LMB at atresia (75-fold). Another intriguing transcript was *nkcc* corresponding to the NKCC protein which transports Na^+^, K^+^ and Cl^-^ into cells and plays a role in maintenance of ionic balance and intracellular pH regulation in human Sertoli cells [43]. To the best of our knowledge, there is no information on the role of this transporter in the fish gonad. Perhaps a decrease in this transcript results in pH changes in atretic oocytes. Some transcripts were also increased, including *glutathione peroxidase 3 isoform 1*, *adenine nucleotide translocator*, *cathepsin B precursor*, and *cell division cycle 42*, among others. Their roles in atresia need to be investigated.

### 4.5 Some Pathways Involved in Oocyte Maturation and Atresia

Many cell-signaling cascades involved in immune response were differentially affected during oocyte maturation. Our data suggest that cell signaling cascades that involve T and B-cell activation, mast cells, and NK cell activation are increased during the early stages of follicular development and are subsequently decreased during AT. Mast cells are located in most tissues and play a key role in the inflammatory process. Mast cells secrete biogenic amines such as histamine and serotonin in response to allergens and cellular injury; these cells also play an important role in the rat ovary during sexual maturation [44]. An interesting role for inflammatory response in fish ovaries during follicular maturation may be in protecting susceptible oocytes from infection [45]. The decrease in these inflammatory pathways during AT may simply reflect that these pathways are no longer needed for oocytes are no longer viable and are being reabsorbed.

An objective of this study was to gain additional insight into the processes that might determine whether an oocyte follows a path towards OV or AT. If energy stores are inadequate, an individual will reduce the number of eggs (via AT) to reabsorb the nutrients. Pathways such as arachidonic acid metabolism and sphingolipid metabolism were involved in OV, while reactive oxygen species metabolism, FSHR -> FOXO1A signaling, TGF signaling, and cholinergic signaling were associated with AT. In addition, oxidative phosphorylation and pathways such as actin cytoskeleton regulation and gap junction regulation were decreased at AT, reflecting changes in energy requirements for atretic oocytes and the breakdown of the germinal vesicle. Although the majority of affected pathways at OV and AT were different, there were some examples of common signalling pathways between OV and AT, such as T-cell pathways and translation control. Interestingly, the fibronectin receptor -> catenin (cadherin-associated protein), beta 1 signalling cascade was down-regulated at both ovulation and atresia while at early growth stages, this pathway is up-regulated and we hypothesize that this pathway plays a role in ooctye maturation in the ovary.

Some other interesting gene expression networks determined to be significantly associated with OM and AT included targets of nerve growth factors (NGF) (increased nearly 110% at ovulation) and expression targets of signal transducers and activators of transcription (STAT5) signaling (decreased 140% during AT). NGF is a protein that stimulates the growth and survival of sympathetic nerve cells. In mammalian ovaries, NGF can be found in the developing oocyte and contributes to the growth and function of sympathetic neurons projecting to the ovaries [46]. In fish, this gene network may be functioning to maintain or enhance neuronal inputs during final oocyte maturation. In contrast, STATs play key roles in growth factor-mediated intracellular signal transduction, proliferation, inflammation, and apoptosis. In the rat ovary, STAT5b and STAT3 signaling pathways show temporal expression patterns during folliculogenesis and luteinization and these pathways are active at different periods to regulate gene expression [47]. STAT5 activates a number of transcription factors and during AT, there may no longer be a need for cell regulation and active DNA transcription.

Gene networks that require more discussion include those controlled by activin/inhibin. Both activin and inhibin are cytokines that are found in the CNS as well as in peripheral tissues. Interestingly, in the ovary, the activin network was significantly higher in expression in earlier stages of oocyte development and decreased towards maturation, but then dramatically increased in ovulated eggs (∼100%). Networks involving *follistatin* (*fst*) and *inhibin B* (*inhb*) on the other hand were significantly decreased during AT (∼130%). A primary role for these cytokine signaling cascades in oocyte maturation in vertebrates are well supported in mammalian studies [48]. These cytokines are involved in both autocrine and paracrine communication between theca and granulosa cells and between oocytes and granulosa cells. In LMB ovulated eggs, the majority of transcripts in the activin network were increased and were related to increases in genes involved in steroid production, lipid transport, and metabolism. Members of the activin network included *fst* and *igf1* and these genes were increased in expression in ovulated eggs.

### 4.6 Seasonal Expression of mPR-alpha, ghr1, lhr, and fshr in the LMB Ovary

Microarray and real-time PCR analysis suggested that LMB *mPR-alpha* mRNA was higher in abundance in secondary growth stages (vitellogenesis) compared to maturation. Progestins are steroidal hormones that include progesterone and 17alpha, 20beta-dihydroxy-4-pregnen-3-one (17α, 20β DHP) that function in regulating gametogenesis. Progestin membrane receptors have been extensively characterized in Atlantic croaker (*Micropogonias undulatus*) [49] and channel catfish (*Ictalurus punctatus*) [50], [51]. In the catfish ovary, *mPR-alpha* is highest immediately preceding the peak in GSI and *lhr*. In contrast, *mPR-beta* has a more constitutive expression in catfish throughout the year and *mPR-gamma* peaks earlier in follicular development and declines in mRNA levels before spawning. In the zebrafish ovary, smaller oocyte sizes showed lower mRNA expression of *mPR-alpha* compared to later stages of development and growth [51]. In goldfish (*Carassius auratus*), *mPR-alpha* mRNA was constitutively expressed during oogenesis and was highest in expression (size of 876–1000 µm) before the maximum size of the oocyte [52]. Thus, data suggest that the progestins play a significant role in early vitellogenesis before final maturation. Interestingly, LMB *mPR-alpha* mRNA expression mirrored more closely the seasonal expression patterns of the catfish *mPR-gamma*. The LMB sequence we obtained is highly homologous to characterized alpha isoforms and the degree in amino acid sequence identity between the α and the β and γ isoforms in catfish is 49% and 30% respectively, thus we are confident that the mPR-alpha isoforms is the isoform being quantified in LMB. Taken together, these studies suggest that there may be differences in mPR isoform expression in teleosts and seasonal expression patterns of all three isoforms need to be further characterized in fishes.

Microarray analysis and real-time PCR identified LMB *ghr* mRNA as higher during primary growth stages than vitellogenic oocytes. This corroborates other studies that show higher levels of *ghr* mRNA early in oocyte development. Using in situ hybridization to detect *ghr* in the ovary of tilapia (*Oreochromis mossambicus*), Kajimura et al. (2004) observed intense staining for *ghr* mRNA in the cytoplasm and nucleus of immature oocytes in the PN stage which suggests an early role for *ghr* during early maturation. Other studies with salmon [53] and trout [54] also indicate higher abundance of the mRNA and activity of Ghr in early ovarian stages. Conversely, both *ghr1* and *ghr2* mRNA expression were highest at sexual maturation compared to regressed stages of reproductive development in tilapia [55]. Thus, there may be differences in expression patterns among fish. In the LMB, only the *ghr1* isoform was investigated and LMB *ghr2* should be investigated to determine whether the two isoforms correspond in expression patterns.

LMB gonadotropin receptor expression was investigated only by real-time PCR experiments since the probes for the two receptors were not present on the microarray. The expression of LMB *lhr and fshr* are comparable to the seasonal patterns observed in other fish, for example female European seabass [25], zebrafish [56], and Atlantic cod [57]. In LMB, it appears that early gonadotropin-induced activation first occurs via FSH stimulation during primary growth stages of LMB oocyte development. This hypothesis is based upon the expression patterns of *fshr* mRNA and the identification of gonadotropin-signaling pathway activation through FSHR signaling by the transcriptomics data in primary growth stages. Of interest, gene networks associated with LH signaling were significantly affected throughout LMB ovarian development. The analysis does not suggest that LH is responsible for these changes, only that downstream targets of LH signaling are increased. This is at a time when the receptor is not yet expressed or is very low, thus the expression network may not yet be responsive to LH and may be regulated by other hormonal factors. Not surprisingly, there was a significant increase in LH expression targets in the ovary at maturation (120%), which corresponds to increased *lhr* mRNA expression associated with ovulation.

### Conclusions

Molecular based studies are needed to better characterize natural variation in ovarian transcriptomics in wild populations of fish. This comprehensive transcriptomics analysis identified genes that are differentially throughout oocyte maturation and atresia. Many environmental pollutants and abiotic factors (e.g. temperature, hypoxia) induce atresia in fish. Therefore, it is important to identify the signaling cascades that correspond to atresia to better understand how these molecular changes relate to gonad morphology. These data suggest that there are distinct transcriptomics fingerprints for LMB ovary stages, and this study provides novel mechanistic insight into molecular signaling cascades underlying oocyte maturation. For example, there may be a role for gene target signaling related to LH in early stages of oocyte growth. This has not been studied to date. Also transcriptomics data suggest that activin/inhibin have a prominent role in regulating DNA replication, steroid metabolism, and lipid transport during final maturation and atresia. Lastly, there appears to be a role for neurotransmitters (e.g. GABA, serotonin, and dopamine), the immune system (T-cell activation, mast cells), cytokines (e.g. activins) and signaling via prostaglandins and arachidonic acid in oocyte development that require further study.

## Supporting Information

File S1 Figure S1: Hierarchical clustering of the transcriptome of each LMB ovarian stage. Clustering was based on all transcriptomics data from the 15 K microarray to investigate overall patterns in gene expression. Primary and secondary growth stages clustered separately as did ovulation and atresia. Figure S2: Heat maps of receptors and transcription factors that showed significant (A) increases with ovulation (B) decreases at ovulation, and (C) and decreases in mRNA abundance with atresia after a time course analysis (SAM). There were no significant transcripts that were induced following atresia, suggesting many processes that are receptor mediated are down regulated with reabsorption of the oocytes. Figure S3: Seasonal dependent expression of (A) *mPR-alpha* (B) *ghr1* (C) *lhr* and (D) *fshr* mRNA. Expression is reported as mean absolute copy number of the transcript ± SEM. Sample sizes/month is n = 4 except for the month of June (n = 3). PG pn (n = 9), PG ca (n = 8), SG eVtg (n = 8), SG lVtg (n = 3), OM (n = 12), and AT (n = 2). Total number of animals used in the stage specific analysis was n = 42. Different letters indicate statistical differences among groups (p<0.05).
(PDF)

File S2 (PDF)

File S3 (XLSX)

File S4 (XLS)

File S5 (DOCX)
