# Supplementary figures and images for: Gene Expression Networks Underlying Ovarian Development in Wild Largemouth Bass (Micropterus salmoides)
Source: PLoS One. 2013 Mar 20;8(3):e59093. doi: 10.1371/journal.pone.0059093 (PMC3604104; doi:10.1371/journal.pone.0059093)

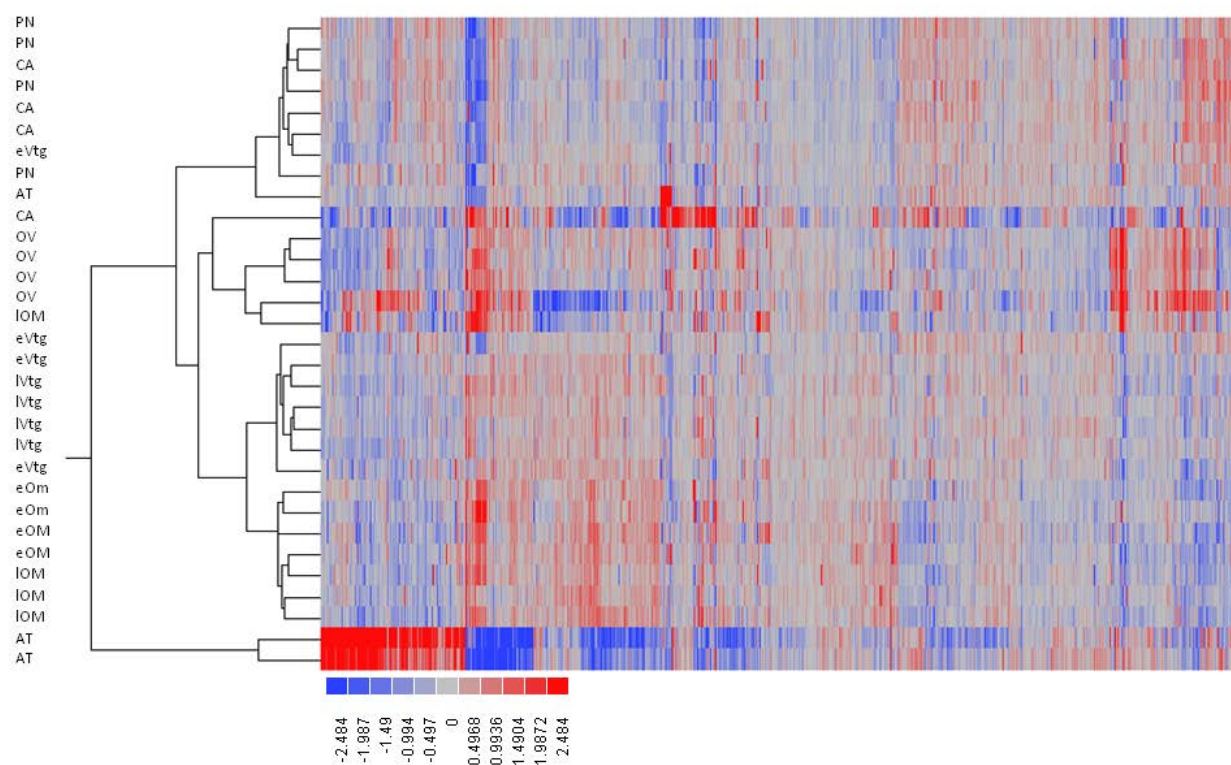

Figure S1

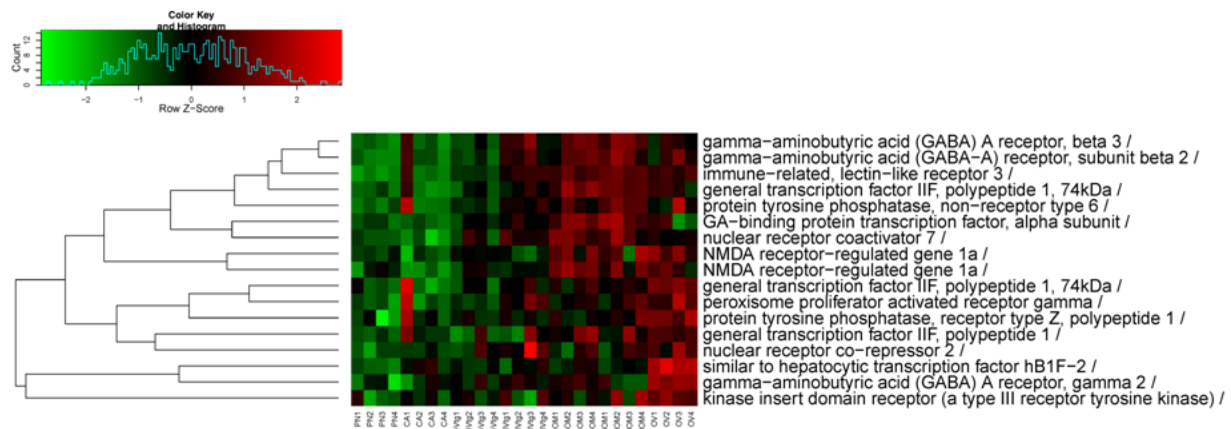

Figure S2A

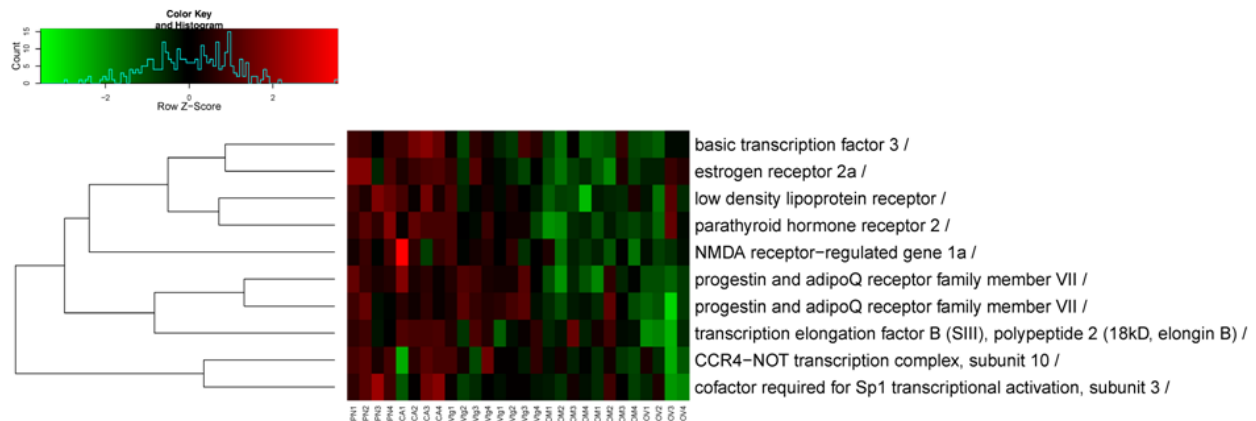

Figure S2B

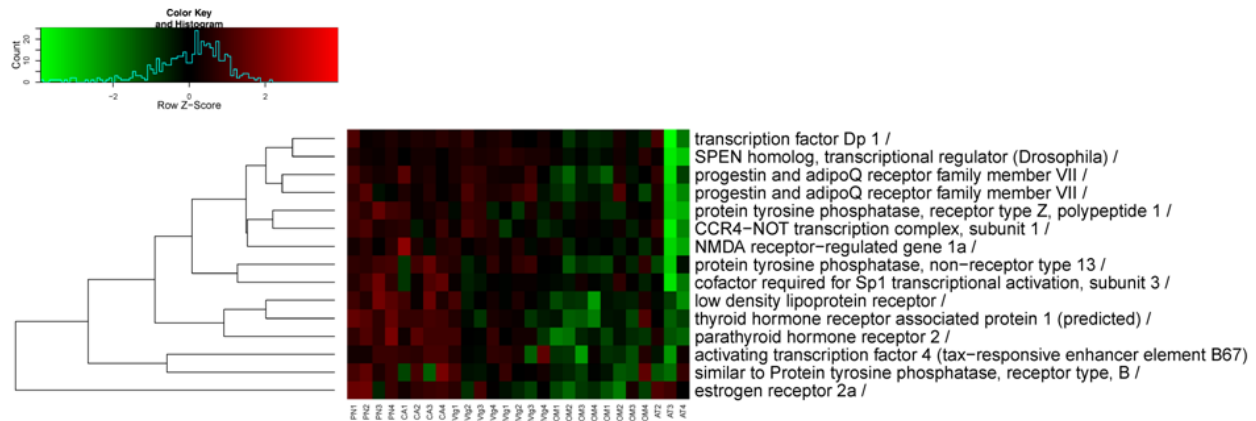

Figure S2C

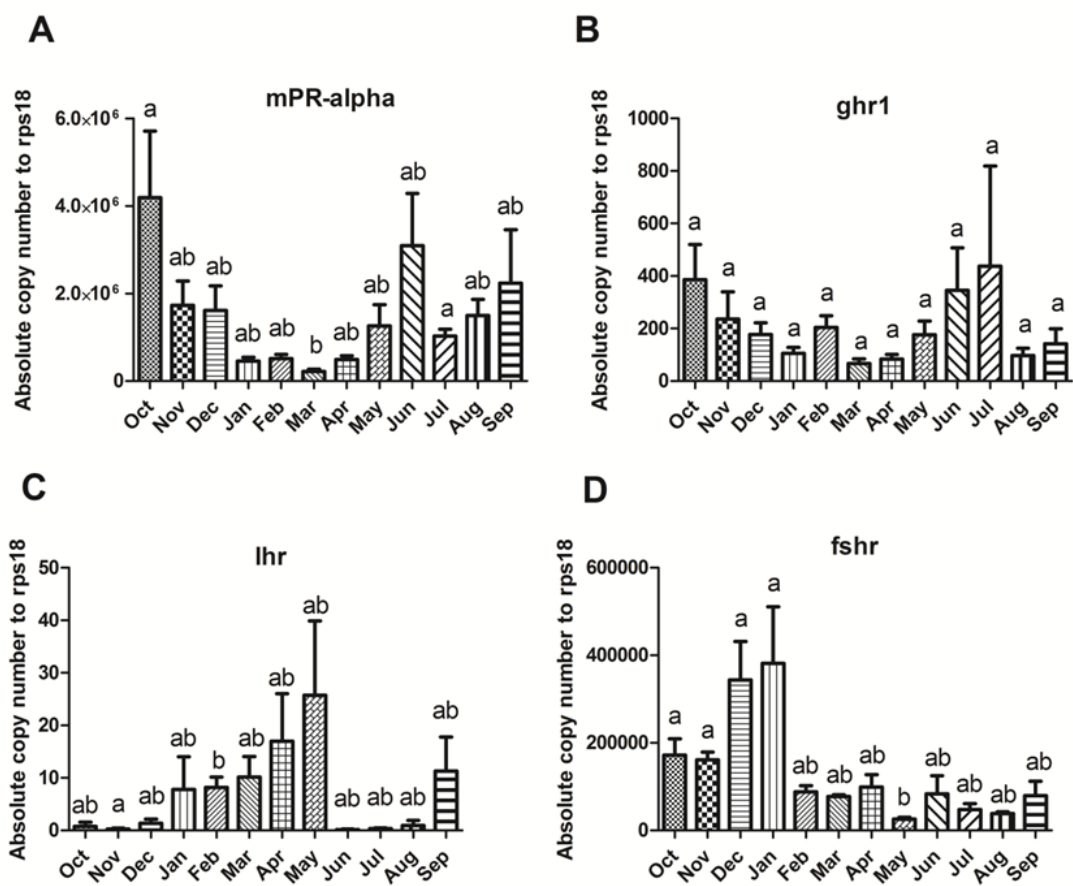

Figure S3

Supplement: File S1 — Figure S1: Hierarchical clustering of the transcriptome of each LMB ovarian stage. Clustering was based on all transcriptomics data from the 15 K microarray to investigate overall patterns in gene expression. Primary and secondary growth stages clustered separately as did ovulation and atresia. Figure S2: Heat maps of receptors and transcription factors that showed significant (A) increases with ovulation (B) decreases at ovulation, and (C) and decreases in mRNA abundance with atresia after a time course analysis (SAM). There were no significant transcripts that were induced following atresia, suggesting many processes that are receptor mediated are down regulated with reabsorption of the oocytes. Figure S3: Seasonal dependent expression of (A) mPR-alpha (B) ghr1 (C) lhr and (D) fshr mRNA. Expression is reported as mean absolute copy number of the transcript ± SEM. Sample sizes/month is n = 4 except for the month of June (n = 3). PG pn (n = 9), PG ca (n = 8), SG eVtg (n = 8), SG lVtg (n = 3), OM (n = 12), and AT (n = 2). Total number of animals used in the stage specific analysis was n = 42. Different letters indicate statistical differences among groups (p<0.05). (PDF) [file pone.0059093.s001.pdf]
