# Supplementary material for: Gene Expression Networks Underlying Ovarian Development in Wild Largemouth Bass (Micropterus salmoides)
Source: PLoS One. 2013 Mar 20;8(3):e59093. doi: 10.1371/journal.pone.0059093 (PMC3604104; doi:10.1371/journal.pone.0059093)
Supplement: File S2 — (PDF) [file pone.0059093.s002.pdf]

**Table S1** Primer sequences used to quantitate (real-time PCR) mRNA steady state levels for LMB receptor. These genes were chosen based upon their significance in LMB reproduction and follicular development. Note that the forward primer is given in the 5' to 3' orientation in relation to the 5' to 3' coding strand (sense) deposited in NCBI GenBank and the reverse primer is 5' to 3' in relation to the complementary strand (antisense).

| Gene | Forward 5'-3' | Reverse 5' to 3' | Amplicon (bp) |
|---|---|---|---|
| | | | |
| *mPR-alpha* | GGG GTC GCT CTC TAC CAA | CAG CAG CCA AAG CAA GTA AG | 137 |
| *ghr1* | AGG GCT CAG ACA CCC AGA | CAG TTT GGG TTT GGA CG | 267 |
| *lhr* | TCA TCC TGC TCT TCA ACG TG | AGC CTT GGT GAA GAT AGC GTA A | 299 |
| *fshr* | CAG TCC TCA TCT TCA CCG ACT T | AGG CGT ACA GGA AGG GGT T | 159 |
| *18S rRNA* | CGG CTA CCA CAT CCA AGG AA | CCT GTA TTG TTA TTT TTC GTC ACT ACC T | 86 |

**Footnote:** All partial gene sequences used in this study have been submitted to GenBank; NCBI gene accession numbers are as follows: LMB *mPR-α,* (FJ030924), LMB *ghr-1* (FJ030934), LMB *lhr* (FJ751229), and LMB *fshr* (FJ751230). The partial gene fragment cloned for *mPR-α* (463 bp) carried 94% nucleotide similarity to the Atlantic croaker sequence (EU095257; *Micropogonias undulates*). Real-time PCR primers were designed to amplify 137 bp of the LMB *mPR-alpha* transcript. The partial gene fragment cloned for LMB *ghr-1* (267 bp) showed 88% nucleotide similarity to that of the Orange spotted grouper (EF052273; *Epinephelus coioides*). The partial gene fragment cloned for LMB *lhr* (299 base pairs; bp) exhibited 91% nucleotide similarity to the European seabass mRNA (AY642114; *Dicentrarchus labrax*), while that for LMB *fshr* (159 bp) showed 95% nucleotide similarity to the mRNA from European seabass (EU282006; *Dicentrarchus labrax*).

**Table S2** Mean fold change of significantly altered mRNA for transcription factors and receptors across ovarian stages based on SAM analysis. Due to the fact that it is not possible to pinpoint at which particular stage a gene significantly changed in expression, the fold changes reported in the table below were determined using a multi-class approach. The fold change is measured in the time course analysis as the difference between the highest and the lowest expression value over all ovarian stages. Duplicate gene titles arise from duplicate probes on the LMB microarray.

| Time Series Analysis | LMB Probe ID | Gene Title | Fold Change |
|---|---|---|---|
| PN_CA_eVtg_lVtg_eOM_lOM_OV | UF_Msa_AF_101575 | general transcription factor IIF, polypeptide 1, 74kDa | 1.661 |
| | UF_Msa_AD_200371 | similar to hepatocytic transcription factor hB1F-2 | 2.545 |
| | UF_Msa_AF_104835 | GA-binding protein transcription factor, alpha subunit | 2.236 |
| | UF_Msa_AF_106495 | general transcription factor IIF, polypeptide 1, 74kDa | 2.094 |
| | UF_Msa_AF_106954 | general transcription factor IIF, polypeptide 1 | 2.264 |
| | UF_Msa_AF_102863 | immune-related, lectin-like receptor 3 | 9.181 |
| | UF_Msa_AF_108143 | gamma-aminobutyric acid (GABA-A) receptor, subunit beta 2 | 2.962 |
| | UF_Msa_AF_107775 | gamma-aminobutyric acid (GABA) A receptor, beta 3 | 2.698 |
| | UF_Msa_AF_101384 | peroxisome proliferator activated receptor gamma | 3.266 |
| | UF_Msa_AD_200114 | protein tyrosine phosphatase, receptor type Z, polypeptide 1 | 3.672 |
| | UF_Msa_AF_103526 | protein tyrosine phosphatase, non-receptor type 6 | 2.823 |

| | | | |
|---|---|---|---|
| | UF_Msa_AF_103274 | nuclear receptor coactivator 7 | 2.163 |
| | UF_Msa_AD_200291 | gamma-aminobutyric acid (GABA) A receptor, gamma 2 | 2.164 |
| | UF_Msa_AF_104357 | NMDA receptor-regulated gene 1a | 1.348 |
| | UF_Msa_AF_106812 | NMDA receptor-regulated gene 1a | 1.381 |
| | UF_Msa_AF_109141 | kinase insert domain receptor (a type III receptor tyrosine kinase) | 1.668 |
| | UF_Msa_AF_104177 | nuclear receptor co-repressor 2 | 2.740 |
| | | | |
| PN_CA_eVtg_lVtg_eOM_lOM_OV | UF_Msa_AF_109021 | basic transcription factor 3 | -1.189 |
| | UF_Msa_AF_101486 | transcription elongation factor B (SIII), polypeptide 2 (18kD, elongin B) | -2.795 |
| | UF_Msa_AF_104468 | cofactor required for Sp1 transcriptional activation, subunit 3 | -2.036 |
| | UF_Msa_AF_104235 | CCR4-NOT transcription complex, subunit 10 | -2.342 |
| | UF_Msa_AD_200191 | progestin and adipoQ receptor family member VII | -2.267 |
| | UF_Msa_AF_104034 | parathyroid hormone receptor 2 | -2.878 |
| | UF_Msa_AD_200271 | estrogen receptor 2a | -3.418 |
| | UF_Msa_AF_109083 | progestin and adipoQ receptor family member VII | -2.354 |
| | UF_Msa_AF_107005 | NMDA receptor-regulated gene 1a | -3.284 |
| | UF_Msa_AD_200112 | low density lipoprotein receptor | -2.113 |

| | | | |
|---|---|---|---|
| PN_CA_eVtg_lVtg_eOM_lOM_AT | UF_Msa_AF_104468 | cofactor required for Sp1 transcriptional activation, subunit 3 | -3.402 |
| | UF_Msa_AF_107712 | transcription factor Dp 1 | -2.919 |
| | UF_Msa_AF_104750 | SPEN homolog, transcriptional regulator (Drosophila) | -6.177 |
| | UF_Msa_AF_101460 | CCR4-NOT transcription complex, subunit 1 | -2.306 |
| | UF_Msa_AF_106226 | activating transcription factor 4 (tax-responsive enhancer element B67) | -1.220 |
| | UF_Msa_AF_104034 | parathyroid hormone receptor 2 | -2.878 |
| | UF_Msa_AD_200321 | protein tyrosine phosphatase, receptor type Z, polypeptide 1 | -8.914 |
| | UF_Msa_AD_200191 | progestin and adipoQ receptor family member VII | -3.756 |
| | UF_Msa_AD_200112 | low density lipoprotein receptor | -2.113 |
| | UF_Msa_AF_100542 | thyroid hormone receptor associated protein 1 (predicted) | -1.818 |
| | UF_Msa_AF_107005 | NMDA receptor-regulated gene 1a | -6.167 |
| | UF_Msa_AF_101308 | similar to Protein tyrosine phosphatase, receptor type, B | -5.548 |
| | UF_Msa_AF_101979 | protein tyrosine phosphatase, non-receptor type 13 | -1.873 |
| | UF_Msa_AD_200271 | estrogen receptor 2a | -3.418 |
| | UF_Msa_AF_109083 | progestin and adipoQ receptor family member VII | -2.524 |

**Table S3** Significant gene ontology for BP for ovarian genes by major stages. Class 0 proportions refer to differentially expressed transcripts and Class 1 proportions refer to transcripts not differentially expressed when transitioning from stage to stage. PG = primary growth (PN and CA), SG = secondary growth (eVtg and lVtg), OM = oocyte maturation (eOM and lOM), OV = ovulation, AT = atresia. Appendix 2 presents all the data from the gene ontology analysis.

| Stage transition | _GO_Biological_Process Category | Raw_p | Class 0 Proportion | Class 1 Proportion | over/under |
|---|---|---|---|---|---|
| PG to SG | GO:0006096; glycolysis | 0.016 | 0.009 | 0.0011 | under |
| PG to SG | GO:0006811; ion transport | 0.019 | 0.012 | 0.0034 | under |
| PG to SG | GO:0006821; chloride transport | 0.045 | 0.005 | 0.0000 | under |
| PG to SG | GO:0008380; RNA splicing | 0.023 | 0.010 | 0.0204 | over |
| SG to OM | GO:0006468; protein amino acid phosphorylation | 0.035 | 0.031 | 0.0147 | under |
| SG to OM | GO:0042157; lipoprotein metabolic process | 0.001 | 0.001 | 0.0110 | over |
| SG to OM | GO:0006810; transport | 0.004 | 0.061 | 0.0971 | over |
| SG to OM | GO:0006118; electron transport | 0.017 | 0.026 | 0.0458 | over |
| SG to OM | GO:0006821; chloride transport | 0.029 | 0.002 | 0.0092 | over |
| SG to OM | GO:0006744; ubiquinone biosynthetic process | 0.039 | 0.004 | 0.0110 | over |
| SG to OM | GO:0006725; aromatic compound metabolic process | 0.040 | 0.002 | 0.0073 | over |
| SG to OM | GO:0006839; mitochondrial transport | 0.040 | 0.002 | 0.0073 | over |

| | | | | | |
|---|---|---|---|---|---|
| SG to OM | GO:0015986; ATP synthesis coupled proton transport | 0.042 | 0.007 | 0.0165 | over |
| OM to OV | GO:0006869; lipid transport | 0.001 | 0.004 | 0.0206 | over |
| OM to OV | GO:0042157; lipoprotein metabolic process | 0.001 | 0.001 | 0.0129 | over |
| OM to OV | GO:0009968; negative regulation of signal transduction | 0.002 | 0.012 | 0.0334 | over |
| OM to OV | GO:0006821; chloride transport | 0.007 | 0.002 | 0.0129 | over |
| OM to OV | GO:0006879; cellular iron ion homeostasis | 0.022 | 0.010 | 0.0257 | over |
| OM to OV | GO:0007420; brain development | 0.034 | 0.004 | 0.0129 | over |
| OM to OV | GO:0006118; electron transport | 0.038 | 0.027 | 0.0463 | over |
| OM to OV | GO:0006826; iron ion transport | 0.042 | 0.010 | 0.0231 | over |
| OM to AT | GO:0007596; blood coagulation | 0.007 | 0.005 | 0.0000 | under |
| OM to AT | GO:0006413; translational initiation | 0.008 | 0.008 | 0.0014 | under |
| OM to AT | GO:0016055; Wnt receptor signaling pathway | 0.013 | 0.004 | 0.0000 | under |
| OM to AT | GO:0042157; lipoprotein metabolic process | 0.013 | 0.004 | 0.0000 | under |
| OM to AT | GO:0042221; response to chemical stimulus | 0.013 | 0.004 | 0.0000 | under |
| OM to AT | GO:0008284; positive regulation of cell proliferation | 0.022 | 0.007 | 0.0014 | under |
| OM to AT | GO:0006869; lipid transport | 0.033 | 0.008 | 0.0021 | under |
| OM to AT | GO:0006826; iron ion transport | 0.037 | 0.015 | 0.0071 | under |

| | | | | | |
|---|---|---|---|---|---|
| OM to AT | GO:0008283; cell proliferation | 0.014 | 0.002 | 0.0085 | over |
| OM to AT | GO:0045039; protein import into mitochondrial inner membrane | 0.020 | 0.000 | 0.0043 | over |
| OM to AT | GO:0006839; mitochondrial transport | 0.036 | 0.001 | 0.0050 | over |
| OM to AT | GO:0007219; Notch signaling pathway | 0.043 | 0.000 | 0.0036 | over |

**Table S4** Gene set enrichment analysis for the major stages in ovarian development. A gene set is shown here if it is altered greater than 20% and the number of members measured in the pathway is greater than 10. Provided in this table are the number of members within a pathway and extended pathway, number of member measured and present on the LMB microarray, normalized enrichment scores, overall change in the pathway (medium fold change) and p-value. No pathway met these criteria for SG to OM transition. The complete list of pathways identified by GSEA is given in appendix 2.

| Stage Transition | Name | Expanded # of Entities | # of Measured Entities | Normalized ES | Median change | p-value |
|---|---|---|---|---|---|---|
| PG to SG | MPL -> SPI1 signaling | 34 | 11 | 1.729 | 1.226 | 0.0000 |
| PG to SG | T-cell receptor -> ATF/CREB signaling | 189 | 16 | 1.775 | 1.229 | 0.0000 |
| PG to SG | FibronectinR -> CTNNB signaling | 38 | 10 | 1.764 | 1.703 | 0.0000 |
| PG to SG | AGER -> NF-kB signaling | 34 | 12 | 1.674 | 1.226 | 0.0041 |
| PG to SG | FibronectinR -> NF-kB signaling | 58 | 14 | 1.734 | 1.293 | 0.0044 |
| PG to SG | T-cell receptor -> NF-kB signaling | 176 | 13 | 1.822 | 1.416 | 0.0045 |
| PG to SG | EGFR/ERBB2 -> HIF1A signaling | 66 | 13 | 1.638 | 1.229 | 0.0136 |
| PG to SG | ICAM2 -> CTNNB/FOXO/STAT3 signaling | 29 | 10 | 1.778 | 1.561 | 0.0138 |
| PG to SG | IL7R -> FOXO/NF-kB signaling | 36 | 10 | 1.693 | 1.469 | 0.0142 |
| PG to SG | B-cell receptor -> NF-kB signaling | 50 | 11 | 1.549 | 1.261 | 0.0167 |
| PG to SG | T-cell receptor -> NFATC signaling | 172 | 10 | 1.638 | 1.469 | 0.0184 |

| | | | | | | |
|---|---|---|---|---|---|---|
| PG to SG | PTAFR -> NF-kB signaling | 63 | 16 | 1.599 | 1.261 | 0.0213 |
| PG to SG | T-cell receptor -> AP-1 signaling | 180 | 15 | 1.649 | 1.261 | 0.0231 |
| PG to SG | GDNF -> HSF1 signaling | 64 | 16 | 1.623 | 1.226 | 0.0250 |
| PG to SG | Notch -> NF-kB signaling | 33 | 10 | 1.609 | 1.261 | 0.0284 |
| PG to SG | AdenosineR -> NF-kB signaling | 49 | 10 | 1.501 | 1.261 | 0.0327 |
| PG to SG | VEGFR -> CTNNB signaling | 27 | 10 | 1.489 | 1.261 | 0.0498 |
| OM to OV | ICAM2 -> CTNNB/FOXO/STAT3 signaling | 29 | 10 | -1.923 | -1.530 | 0.0000 |
| OM to OV | T-cell receptor -> NFATC signaling | 172 | 10 | -1.876 | -1.530 | 0.0000 |
| OM to OV | TLR1/2/6 -> NF-kB signaling | 39 | 12 | -1.655 | -1.390 | 0.0000 |
| OM to OV | T-cell receptor -> ATF/CREB signaling | 189 | 16 | -1.810 | -1.402 | 0.0044 |
| OM to OV | FibronectinR -> CTNNB signaling | 38 | 10 | -1.792 | -1.496 | 0.0046 |
| OM to OV | T-cell receptor -> NF-kB signaling | 176 | 13 | -1.719 | -1.474 | 0.0047 |
| OM to OV | Sphingolipid metabolism | 164 | 27 | -1.631 | -1.399 | 0.0049 |
| OM to OV | T-cell receptor -> CREBBP signaling | 176 | 11 | -1.744 | -1.541 | 0.0088 |
| OM to OV | IL7R -> FOXO/NF-kB signaling | 36 | 10 | -1.549 | -1.474 | 0.0226 |
| OM to OV | ErythropoietinR -> NF-kB signaling | 48 | 10 | -1.551 | -1.368 | 0.0233 |
| OM to OV | NK Cell Activation | 523 | 74 | -1.458 | -1.236 | 0.0280 |

| | | | | | | |
|---|---|---|---|---|---|---|
| OM to OV | FibronectinR -> NF-kB signaling | 58 | 14 | -1.570 | -1.357 | 0.0313 |
| OM to OV | Notch -> NF-kB signaling | 33 | 10 | -1.530 | -1.298 | 0.0320 |
| OM to OV | ThrombinR -> NF-kB signaling | 32 | 11 | -1.550 | -1.474 | 0.0359 |
| OM to OV | AdrenergicRb -> STAT3 signaling | 75 | 13 | -1.514 | -1.368 | 0.0417 |
| OM to AT | Gap Junction Regulation | 639 | 57 | -1.946 | -2.074 | 0.0000 |
| OM to AT | Skeletal Myogenesis Control | 569 | 69 | -1.825 | -1.565 | 0.0000 |
| OM to AT | Actin Cytoskeleton Regulation | 473 | 86 | -1.830 | -1.454 | 0.0000 |
| OM to AT | Guanylate Cyclase Pathway | 1219 | 198 | -1.763 | -1.327 | 0.0000 |
| OM to AT | Translation Control | 984 | 213 | 1.288 | 1.207 | 0.0000 |
| OM to AT | Respiratory chain and oxidative phosphorylation | 118 | 42 | 1.516 | 1.486 | 0.0000 |
| OM to AT | ROS metabolism | 74 | 14 | 1.880 | 1.680 | 0.0000 |
| OM to AT | T-cell receptor -> AP-1 signaling | 180 | 15 | -1.640 | -1.576 | 0.0036 |
| OM to AT | ICAM2 -> CTNNB/FOXO/STAT3 signaling | 29 | 10 | -1.677 | -2.930 | 0.0036 |
| OM to AT | T-cell receptor -> ATF/CREB signaling | 189 | 16 | -1.543 | -1.409 | 0.0037 |
| OM to AT | Gonadotrope Cell Activation | 698 | 102 | -1.490 | -1.324 | 0.0053 |
| OM to AT | EphrinR -> actin signaling | 216 | 42 | -1.604 | -1.217 | 0.0058 |
| OM to AT | Focal Adhesion Regulation | 308 | 45 | -1.587 | -1.343 | 0.0059 |

| | | | | | | |
|---|---|---|---|---|---|---|
| OM to AT | UrokinaseR -> ELK-SRF signaling | 58 | 13 | -1.574 | -1.474 | 0.0068 |
| OM to AT | ERBB2/3 -> EP300/ETS/ETV/SP1 signaling | 63 | 13 | -1.616 | -1.474 | 0.0104 |
| OM to AT | T-cell receptor -> NFATC signaling | 172 | 10 | -1.635 | -1.474 | 0.0105 |
| OM to AT | VEGFR -> CTNNB signaling | 27 | 10 | -1.576 | -2.305 | 0.0109 |
| OM to AT | Adherens Junction Regulation | 692 | 51 | -1.559 | -1.517 | 0.0142 |
| OM to AT | EGFR/ERBB2 -> HIF1A signaling | 66 | 13 | -1.562 | -1.474 | 0.0179 |
| OM to AT | PTAFR -> NF-kB signaling | 63 | 16 | -1.598 | -1.474 | 0.0181 |
| OM to AT | Mannose metabolism | 73 | 18 | 1.702 | 1.371 | 0.0192 |
| OM to AT | SerotoninR1 -> FOS signaling | 109 | 19 | -1.543 | -1.343 | 0.0196 |
| OM to AT | FSHR -> FOXO1A signaling | 54 | 14 | -1.622 | -1.474 | 0.0211 |
| OM to AT | CholinergicRm -> CREB/ELK-SRF signaling | 107 | 18 | -1.599 | -1.343 | 0.0230 |
| OM to AT | T-cell receptor -> CREBBP signaling | 176 | 11 | -1.503 | -2.164 | 0.0251 |
| OM to AT | GFR -> NCOR2 signaling | 130 | 18 | -1.621 | -1.474 | 0.0264 |
| OM to AT | GRM1/5 -> CREB signaling | 110 | 13 | -1.594 | -2.074 | 0.0276 |
| OM to AT | AdrenergicRb -> STAT3 signaling | 75 | 13 | -1.494 | -1.474 | 0.0285 |
| OM to AT | T-cell receptor -> NF-kB signaling | 176 | 13 | -1.527 | -1.531 | 0.0289 |
| OM to AT | FibronectinR -> CTNNB signaling | 38 | 10 | -1.526 | -1.890 | 0.0320 |

| | | | | | | |
|---|---|---|---|---|---|---|
| OM to AT | VasopressinR1 -> MEF/MYOD/NFATC/MYOG signaling | 119 | 23 | -1.504 | -1.447 | 0.0325 |
| OM to AT | Mast Cell Activation | 529 | 83 | -1.418 | -1.300 | 0.0392 |
| OM to AT | GNRHR -> ELK-SRF signaling | 77 | 13 | -1.499 | -2.056 | 0.0473 |
| OM to AT | GFR -> AP-1/CREB/CREBBP/ELK-SRF/MYC signaling | 156 | 26 | -1.496 | -1.454 | 0.0497 |
| OM to AT | NK Cell Activation | 523 | 74 | -1.389 | -1.301 | 0.0500 |
