# Supplementary material for: Gene Expression Networks Underlying Ovarian Development in Wild Largemouth Bass (Micropterus salmoides)
Source: PLoS One. 2013 Mar 20;8(3):e59093. doi: 10.1371/journal.pone.0059093 (PMC3604104; doi:10.1371/journal.pone.0059093)
Supplement: File S5 — (DOCX) [file pone.0059093.s005.docx]

**Abbreviation List**

A2M- alpha-2-macroglobulin

ADCYAP1R1- adenylate cyclase activating polypeptide 1 (pituitary) receptor type I

AKT1- v-akt murine thymoma viral oncogene homolog 1

ANXA5- Annexin A5

APOA4- apolipoprotein A-IV

AR, androgen receptor

BCL2L1- BCL2-like 1

cRNA – complementary RNA

CASP3- caspase 3, apoptosis-related cysteine peptidase

CCNA2- cyclin A2

CCND2- cyclin D2

CCNE1- cyclin E1

CD69- CD69 molecule

CDH2- cadherin 2, type 1, N-cadherin (neuronal)

CDK2- cyclin-dependent kinase 2

CGA- glycoprotein hormones, alpha polypeptide

CREB1- cAMP responsive element binding protein 1

CTNNB1- catenin (cadherin-associated protein), beta 1, 88kDa

CYP19A1- cytochrome P450, family 19, subfamily A, polypeptide 1

CYP3A4- cytochrome P450, family 3, subfamily A, polypeptide 4

DUSP1- dual specificity phosphatase 1

E2 – estrogen

ELISA - Enzyme-linked immunosorbent assay

ESR1- estrogen receptor 1

FABP1- fatty acid binding protein 1, liver

FASN- fatty acid synthase

FDR – False Discovery Rate

FN1- fibronectin 1

FOS- v-fos FBJ murine osteosarcoma viral oncogene homolog

FOXC1, forkhead box C1

FOXO1 - forkhead box O1

FSHB- follicle stimulating hormone, beta polypeptide

FSHR - follicle stimulating hormone receptor

FST- follistatin

GAD1- glutamate decarboxylase 1 (brain, 67kDa)

GAD2- glutamate decarboxylase 2 (pancreatic islets and brain, 65kDa)

GABA – gamma-aminobutyric acid

GH1/ GHR - growth hormone 1 / growth hormone receptor

GNRHR- gonadotropin-releasing hormone receptor

GOI – gene of interest

GSK3B- glycogen synthase kinase 3 beta

GSEA – gene set enrichment analysis

GSI – gonadosomatic index

GVBD – germinal vesicle breakdown

HBE1- hemoglobin, epsilon 1

HBZ- hemoglobin, zeta

HSP90AA1- heat shock protein 90kDa alpha (cytosolic), class A member 1

IGF1- insulin-like growth factor 1 (somatomedin C)

IgG - Immunoglobulin G

INHBA- inhibin, beta A

ITGB1- integrin, beta 1 (fibronectin receptor, beta polypeptide, antigen CD29 includes MDF2, MSK12)

KCNC1- potassium voltage-gated channel, Shaw-related subfamily, member 1

KDR- kinase insert domain receptor (a type III receptor tyrosine kinase)

KEGG - Kyoto Encyclopedia of Genes and Genomes

KPNA4- karyopherin alpha 4 (importin alpha 3)

LMB – Largemouth Bass

LDLR- low density lipoprotein receptor

LH / LHR- luteinizing hormone and luteinizing hormone receptor

LHB- luteinizing hormone beta polypeptide

MCL1- myeloid cell leukemia sequence 1 (BCL2-related)

mPR-alpha – membrane progestin receptor alpha

MYH10- myosin, heavy chain 10, non-muscle

MYOD1, myogenic differentiation 1

NOTCH1- Notch homolog 1, translocation-associated (Drosophila)

NR0B1- nuclear receptor subfamily 0, group B, member

NRN1- neuritin 1

PDPK1- 3-phosphoinositide dependent protein kinase-1

PI3K- phosphatidylinositol 3-kinase

PMTs – photomultiplier tubes

POMC- proopiomelanocortin

PPARG- peroxisome proliferator-activated receptor gamma

PRL- prolactin

SAM – significance of microarray analysis

SCARB1- scavenger receptor class B, member 1

SDHC- succinate dehydrogenase complex, subunit C, integral membrane protein, 15kDa

SNEA – sub-network enrichment analysis

SPARC- secreted protein, acidic, cysteine-rich (osteonectin)

STAR- steroidogenic acute regulatory protein

T - testosterone

TGF-beta – transforming growth factor beta

TF- transferring

TH- tyrosine hydroxylase

VEGFA- vascular endothelial growth factor A

VTG - vitellogenin
